# Pharmacological Chaperones and Coenzyme Q_10_ Treatment Improves Mutant β-Glucocerebrosidase Activity and Mitochondrial Function in Neuronopathic Forms of Gaucher Disease

**DOI:** 10.1038/srep10903

**Published:** 2015-06-05

**Authors:** Mario de la Mata, David Cotán, Manuel Oropesa-Ávila, Juan Garrido-Maraver, Mario D. Cordero, Marina Villanueva Paz, Ana Delgado Pavón, Elizabet Alcocer-Gómez, Isabel de Lavera, Patricia Ybot-González, Ana Paula Zaderenko, Carmen Ortiz Mellet, José M. García Fernández, José A. Sánchez-Alcázar

**Affiliations:** 1 Centro Andaluz de Biología del Desarrollo (CABD-CSIC-Universidad Pablo de Olavide), and Centro de Investigación Biomédica en Red: Enfermedades Raras, Instituto de Salud Carlos III, Sevilla; 2 Facultad de Odontología, Universidad de Sevilla, Sevilla; 3 Instituto de Biomedicina de Sevilla (IBIS)-CSIC, Hospital Virgen del Rocío, Sevilla 41013.; 4 Sistemas Físicos, Químicos y Naturales-Universidad Pablo de Olavide; 5 Dept. Química Orgánica, Facultad de Química, Universidad de Sevilla, Sevilla; 6 Instituto de Investigaciones Químicas (IIQ) CSIC-Universidad de Sevilla, Sevilla, Spain

## Abstract

Gaucher disease (GD) is caused by mutations in the GBA1 gene, which encodes lysosomal β-glucocerebrosidase. Homozygosity for the L444P mutation in GBA1 is associated with high risk of neurological manifestations which are not improved by enzyme replacement therapy. Alternatively, pharmacological chaperones (PCs) capable of restoring the correct folding and trafficking of the mutant enzyme represent promising alternative therapies.Here, we report on how the L444P mutation affects mitochondrial function in primary fibroblast derived from GD patients. Mitochondrial dysfunction was associated with reduced mitochondrial membrane potential, increased reactive oxygen species (ROS), mitophagy activation and impaired autophagic flux.Both abnormalities, mitochondrial dysfunction and deficient β-glucocerebrosidase activity, were partially restored by supplementation with coenzyme Q_10_ (CoQ) or a L-idonojirimycin derivative, N-[N’-(4-adamantan-1-ylcarboxamidobutyl)thiocarbamoyl]-1,6-anhydro-L-idonojirimycin (NAdBT-AIJ), and more markedly by the combination of both treatments. These data suggest that targeting both mitochondria function by CoQ and protein misfolding by PCs can be promising therapies in neurological forms of GD.

Lysosomal storage diseases (LSDs) describe a heterogeneous group of rare inherited metabolic disorders which result from the absence or loss of function of lysosomal proteins and characterized by the progressive accumulation of undigested material in lysosomes. The accumulation of substances affects the function of lysosomes and other organelles, resulting in secondary changes, such as impairment of autophagy, mitochondrial dysfunction and inflammation^1^, which ultimately results in cellular defects and clinical abnormalities. LSDs frequently involve the central nervous system (CNS), where neuronal dysfunction or loss results in progressive motor degeneration and premature death.

Gaucher disease (GD), the LSD with the highest prevalence, is caused by mutations in the GBA1 gene that results in defective and insufficient activity of the enzyme β-glucocerebrosidase (GCase). Decreased catalytic activity and/or instability of GCase leads to accumulation of glucosylceramide (GlcCer) and glucosylsphingosine (GlcSph) in the lysosomes of macrophage cells and visceral organs. GD can be subdivided into 3 types based on age at onset and neurological manifestations. Gaucher patients without CNS involvement are classified as type I, while those with CNS involvement are type II or type III^2^. The disease manifests itself in a wide range of organ systems, with signs of hepatosplenomegaly, osteopenia, anemia, cardiopulmonary disease, or neurodegenerative syndromes. Neurological signs include ataxia, pyramidal signs, myoclonic epilepsy, or supra nuclear ophthalmoplegia^3^. Additionally, mutations in the GBA1 gene are a risk factor for Parkinson’s disease and other dementia with Lewy bodies^4^,^5^, as it appears that similar underlying defects in autophagy and mitochondrial dysfunction may link the pathophysiology of these disorders^6^. Lysosomes are essential for autophagy, and autophagic clearance of dysfunctional mitochondria and represent an important element of mitochondrial quality control. Given that cellular quality control is essential to maintaining correct mitochondrial function, it can be inferred that dysfunction of this organelle in LSDs correlates with abnormal accumulation of mitochondria, mitochondrial dysfunction and a block of autophagy^7^.

Currently, enzyme replacement therapy (ERT) and small-molecule substrate reduction therapy (SRT) are the only approved treatment options for patients with the non-neurologic form of GD. ERT, based on the intravenous administration of recombinant GCase, is the effective treatment for type I GD. However, the CNS manifestations of type II and III GD do not respond well to ERT due to the inability of exogenous enzyme to cross the blood-brain barrier (BBB)^8^. SRT drugs have the potential for better CNS penetration and producing some neurological benefit as the therapeutic agent is a small molecule, such as N-butyl-1-deoxynojirimycin (NB-DNJ, Miglustat, Zavesca®), which acts as a weak inhibitor of glucosylceramide synthase, thus reducing the biosynthesis of GlcCer. Miglustat has been approved for use in patients with mild-to-moderate type I Gaucher disease, and is currently being evaluated in neuronopathic Gaucher patients, though a recent report showed no significant benefit for the neurological manifestations of type III patients^9^. Furthermore, many patients treated with Miglustat have experienced side effects including diarrhea, weight loss, tremor, and peripheral neuropathy^10^.

More recently, pharmacological chaperone therapy (PCT) has been proposed as a potential treatment for GD^11^ . PCs are designed to selectively bind to and stabilize the folded state of a given LSD-associated enzyme in the endoplasmic reticulum (ER). In the case of GD, PCs are designed for facilitating proper folding, trafficking to lysosomes and function of the mutant GCase^12^. PCs also have the potential to attenuate the unfolded protein response and prevent ER stress that can lead to apoptosis and other inflammatory responses^13^. The fact that PCs are less expensive, can be given orally and usually cross the BBB, opens up the possibility of treating Type II and Type III GD patients with neurological involvement that are not responsive to ERT.

One of the most prevalent GD-causing mutations in humans is a L444P missense mutation in the GCase protein, which results in its disrupted folding in the ER and impaired post-ER trafficking. Patients homozygous for the L444P mutation usually display a more severe neurologic form of GD^14^. The fact that this mutation is located in a non-catalytic domain of GCase makes the protein particularly refractory to the rescuing action of active-site directed pharmacological chaperones, with only a few examples on record^15^,^16^,^17^. Evaluation of new bicyclic L-idonojirimycin derivatives related to the calystegine alkaloids^18^,^19^ as PCs in human GD fibroblasts homozygous for the L444P mutation, let identify some representatives bearing potential therapeutic options for increasing GCase activity and enzyme trafficking to lysosomes in GD patients with the L444P/L444P genotype 20. These new chaperones belong to the sp^2^-iminosugar glycomimetics, characterized by a high selectivity among lysosomal glycosidases^21^,^22^, and can be prepared through efficient reaction sequences with relatively low synthetic cost^23^. One of the most promising candidates is *N*-[*N’*-(4-adamantan-1-ylcarboxamidobutyl)thiocarbamoyl]-1,6-anhydro-L-idonojirimycin (NAdBT-AIJ)^20^.

Likewise, as mitochondrial dysfunction and/or impaired mitochondria elimination may be associated to alterations of lysosome-dependent processes, coenzyme Q_10_ (CoQ), an antioxidant and mitochondrial energizer, is currently considered as a potential experimental drug for the treatment of neurodegenerative diseases in general^24^ and lysosomal diseases in particular^25^. We conceived that addressing both mutant GCase rescuing by NAdBT-AIJ as PC and mitochondrial function by CoQ might lead to beneficial synergies.

The aim of our study was to assess mitochondrial dysfunction in Gaucher fibroblasts harboring the L444P mutant GCase and elucidate whether the single or combined treatments with CoQ and PC NadBT-AIJ were able to ameliorate their biochemical phenotype.

## Results

### Effect of CoQ and PC NAdBT-AIJ Supplementation on Mitochondrial Dysfunction in Gaucher Fibroblasts

**Figure 1:**
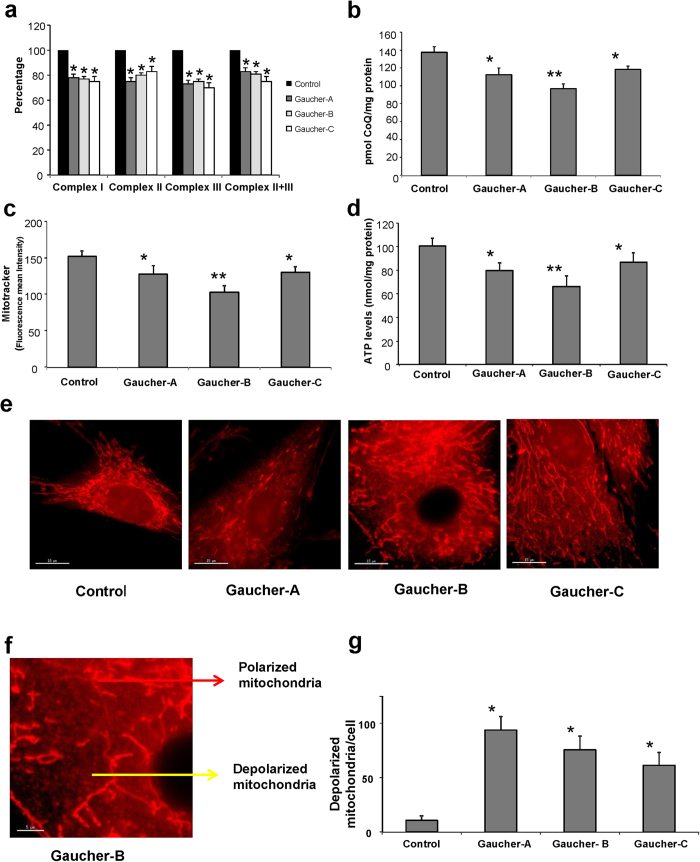
Mitochondrial dysfunction in Gaucher fibroblasts. **(a)** Mitochondrial enzymatic activities of complex I, II, III and II+III in control and Gaucher fibroblasts was determined as described in Materials and Methods. **(b)** CoQ levels in control and Gaucher fibroblasts were determined by hexane extraction and HPLC separation as described in Materials and Methods. **(c)** Mitochondrial membrane potential (ΔΨm) was assessed by flow cytometry using MitoTracker Red. A clear decrease of ΔΨm was observed in Gaucher fibroblasts. **(d)** ATP levels in control and Gaucher fibroblasts. A significant decrease of ATP levels was observed in Gaucher fibroblasts **(e)** Representative images of MitoTracker staining in control and Gaucher fibroblasts. Scale bar=15 μm. **(f)** Magnification of a small area in a Gaucher fibroblast. Red arrow indicates tubular mitochondria with normal polarization. Yellow arrow indicates small mitochondria with a striking depolarization in Gaucher fibroblasts. Scale bar=5 μm. **(g)** Quantification of depolarized mitochondria in control and Gaucher fibroblasts by fluorescence microscopy determined from 100 randomly selected cells by fluorescence imaging analysis. For control cells, the data are the mean±SD for experiments conducted on 3 different control cell lines. Data represent the mean±SD of 3 separate experiments. *p < 0.05 between control and Gaucher fibroblasts. ^**^p < 0.01 between control and Gaucher fibroblasts.

To determine the presence of mitochondrial dysfunction in Gaucher fibroblasts harboring the L444P/L444P mutation, we first measured the activities of respiratory chain enzymes in control and patient (Gaucher A, B and C) fibroblasts. Complex I, II, III and II+III activities were reduced by ~20% in Gaucher A, Gaucher B and Gaucher C fibroblasts respect to controls (Fig. 1a). Given the critical role of CoQ in mitochondria function, transferring reducing equivalents from complexes I and II to complex III, it has been suggested that CoQ levels could be a useful biological marker of mitochondrial function^26^. Therefore, we next assessed whether fibroblasts derived from the patients with GD were deficient in CoQ. The average CoQ content of the three Gaucher fibroblasts cell lines was reduced by 24% relative to the average value of three control fibroblasts (Fig. 1b).

To assess the functional consequences of reduced respiratory chain enzyme activities and CoQ levels in Gaucher fibroblasts, we determined the mitochondrial membrane potential (ΔΨm) in both control and Gaucher fibroblasts by MitoTracker staining coupled with flow cytometry analysis. As shown in Fig 1c, Gaucher fibroblasts showed a significant reduction in ΔΨm, suggesting that the capacity to produce ATP may be diminished in Gaucher fibroblasts. Mitochondrial depolarization was also confirmed by measuring the ratio of TMRM (tetramethylrhodamine methyl ester) to MTG (MitoTracker Green FM) signal by flow cytometry (Supplementary Figure S1a).

Indeed, ATP levels were reduced to 30% of the control value in Gaucher fibroblasts (Fig. 1d). MitoTracker staining and imaging analysis of individual mitochondria confirmed the presence of normal tubular mitochondria (Fig. 1e,f,g, red arrow) along with a population of small rounded mitochondria with a striking depolarization (Fig. 1e,f,g; yellow arrow).

**Figure 2:**
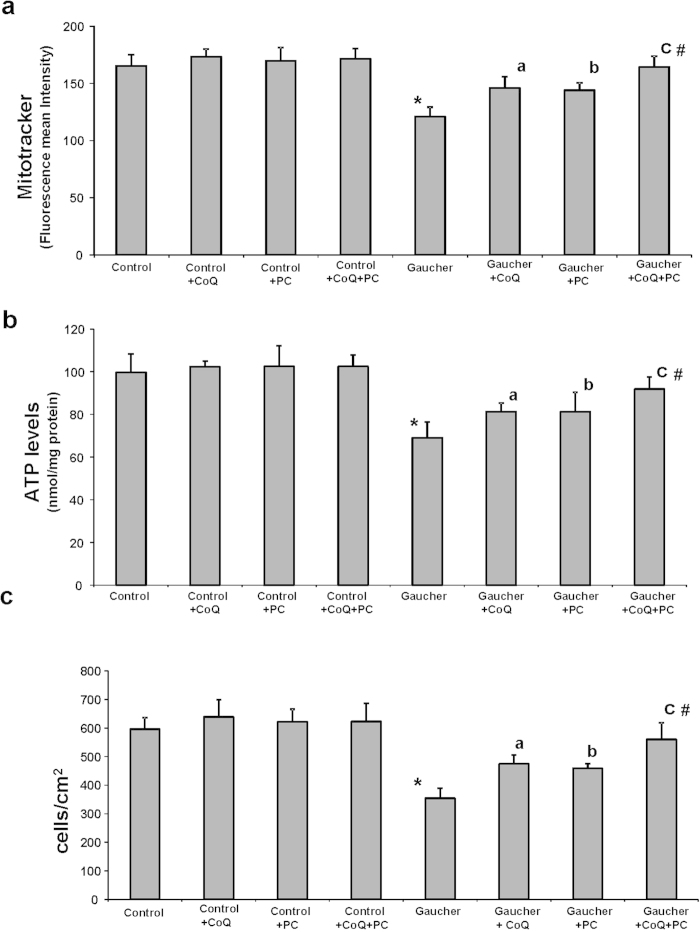
Mitochondrial function in Gaucher fibroblasts is recovered by CoQ and PC NAdBT-AIJ treatments. Control and Gaucher-B fibroblasts were cultured in the absence or presence of CoQ (25 μM), PC NAdBT-AIJ (25 μM) or CoQ + PC (25 μM + 25 μM) for 96 h. **(a)** Mitochondrial membrane potential (ΔΨm) in control and Gaucher fibroblasts. ΔΨm was assessed by flow cytometry using MitoTracker Red staining. **(b)** Adenosine-5’-triphosphate (ATP) levels in control and Gaucher fibroblasts. **(c)** Cell proliferation of control and Gaucher fibroblasts. For the control cells the data are the means±SD of three different control cell lines. Data represent the mean±SD of 3 separate experiments. *p < 0.01 between control and Gaucher fibroblasts. ^a^p < 0.05 between the presence and the absence of CoQ. ^b^p < 0.05 between the presence and the absence of PC. ^c^p < 0.05 between the presence and the absence of CoQ+PC. ^#^p < 0.05 between CoQ+PC and CoQ or PC treatment.

To elucidate whether bioenergetic impairment in Gaucher fibroblasts could be attenuated by improving mitochondrial function by CoQ and restoring GCase folding by PC administration, we quantified ΔΨm and ATP levels after CoQ (25 μM), PC NAdBT-AIJ (25 μM) or CoQ + PC (25 μM + 25 μM) supplementation. Both CoQ or PC treatments and specially the combined treatment of CoQ + PC resulted in a significant increase in ΔΨm and cellular ATP levels in Gaucher fibroblasts, but it had no effect in control cells (Fig. 2b). CoQ + PC effectiveness in improving ΔΨm and ATP levels was confirmed in the three Gaucher cell lines (Supplementary Figure S1b and S1c).

Next, we determined the effect of the L444P/L444P GCase mutation on cellular proliferation, as an indicator of correct or altered cell homeostasis, after CoQ and/or PC treatments. Proliferation rate was increased in Gaucher fibroblasts after both CoQ or PC treatments, and more significantly under the combined treatment CoQ + PC (Fig. 2c).

Staining with MitoTracker Red and cytochrome c immunostaining revealed discrete staining of mitochondria in control cells, with mitochondria organized in a tubular network, whereas small, rounded depolarized mitochondria were observed in Gaucher fibroblasts (Supplementary Figure S2a and S2b). The fact that Mitotracker staining colocalized with cytochrome c, a mitochondrial marker, (Supplentary Figure S2a) confirmed the specificity of MitoTracker staining and verified that mitochondrial depolarization in Gaucher fibroblasts was not a result of cytochrome c release, as occurs in apoptosis^27^. Supplementation with CoQ (25 μM) or PC NAdBT-AIJ (25 μM) and specially the combined treatment of both CoQ + PC restored normal mitochondrial network and MitoTracker Red uptake in Gaucher fibroblasts, indicating restoration of ΔΨm (Figure 2Sa and 2Sb). Indeed, determination of GlcCer levels and ΔΨm in control and Gaucher fibroblast cultures after the incubation with conduritol B-epoxide (CBE), an irreversible inhibitor of GCase, or exogenous GlcCer unequivocally established that accumulation of GlcCer induced a decrease in ΔΨm (Supplementary Figure S3a, S3b and S3c), strongly suggesting that the effects of GlcCer accumulation extend beyond the lysosomal compartment and affect other cellular organelles such as mitochondria.

### Effect of CoQ and PC NAdBT-AIJ on Reactive Oxygen Species (ROS) Production in Gaucher Fibroblasts

**Figure 3:**
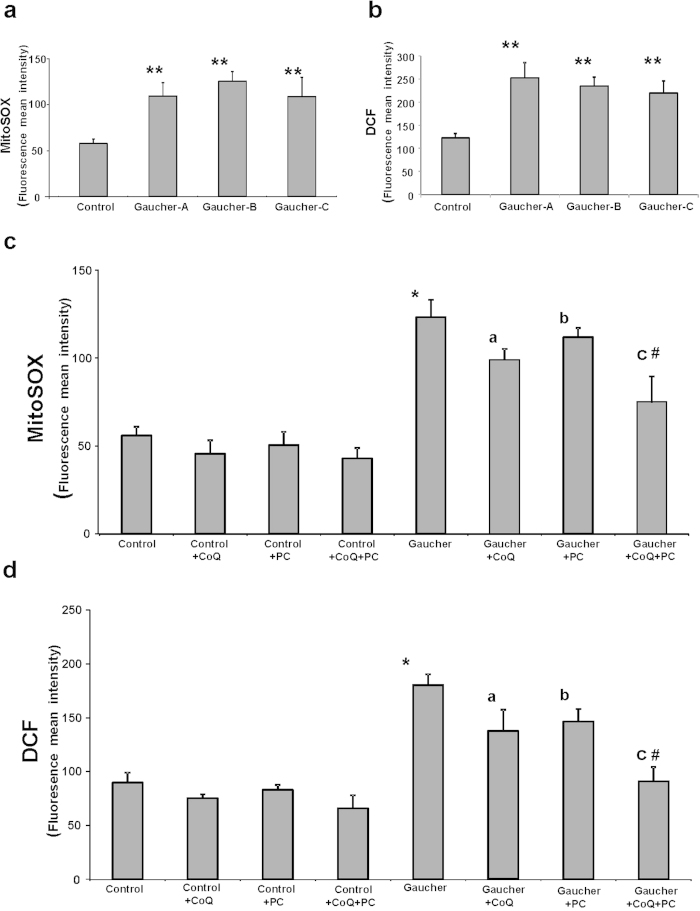
ROS production in Gaucher fibroblasts. **(a)** Mitochondrial ROS levels in control and Gaucher fibroblasts. Results are expressed as the ratio of MitoSOX signal to 10-N-nonyl acridine orange signal. **(b)** H_2_O_2_ levels in control and Gaucher fibroblasts by CMH_2_-DCFDA staining coupled with flow cytometry analysis. **(c)** Mitochondrial ROS levels in control and Gaucher-B fibroblasts cultured, in the absence or presence of CoQ (25 μM), PC NAdBT-AIJ (25 μM) or the combination of both CoQ + PC (25 μM + 25 μM) for 96 h. **(d)** H_2_O_2_ levels in control and Gaucher-B fibroblasts cultured, in the absence or presence of CoQ (25 μM), PC NAdBT-AIJ (25 μM) and the combination of both CoQ + PC (25 μM + 25 μM) for 96 h. The mean ± SD of 3 independent experiments are showed. *p < 0.01 between control and Gaucher fibroblasts. ^a^p < 0.05 between the presence and the absence of CoQ. ^b^p < 0.05 between the presence and the absence of PC. ^c^p < 0.05 between the presence and the absence of CoQ + PC. ^#^p < 0.05 between CoQ + PC and CoQ or PC treatment.

It is well established that mitochondrial dysfunction is associated with increased ROS production. Therefore, we examined mitochondrial ROS levels using the mitochondrial superoxide indicator MitoSOX Red in control and L444P Gaucher fibroblasts. At the same time, we estimated mitochondrial mass with 10-N-nonyl acridine orange (NAO) and determined the ratio of MitoSOX signal to NAO fluorescence. H_2_O_2_ levels were also measured using CMH_2_-DCFDA and flow cytometry analysis. Mitochondrial superoxide production and H_2_O_2_ levels were increased approximately by 2.5-fold (Fig. 3a,b) and by 2-fold respectively, suggesting increased oxidative stress in Gaucher fibroblasts. Supplementation with CoQ (25 μM), PC NAdBT-AIJ (25 μM) and specially the combined treatment of both CoQ + PC induced a considerable reduction in mitochondrial superoxide and H_2_O_2_ levels in Gaucher cultures, but had no effect in control cultures (Fig. 3c,d). CoQ + PC effectiveness in reducing ROS levels was confirmed in the three Gaucher cell lines (Supplementary Figure S4).

### Autophagy in Gaucher Fibroblasts

**Figure 4:**
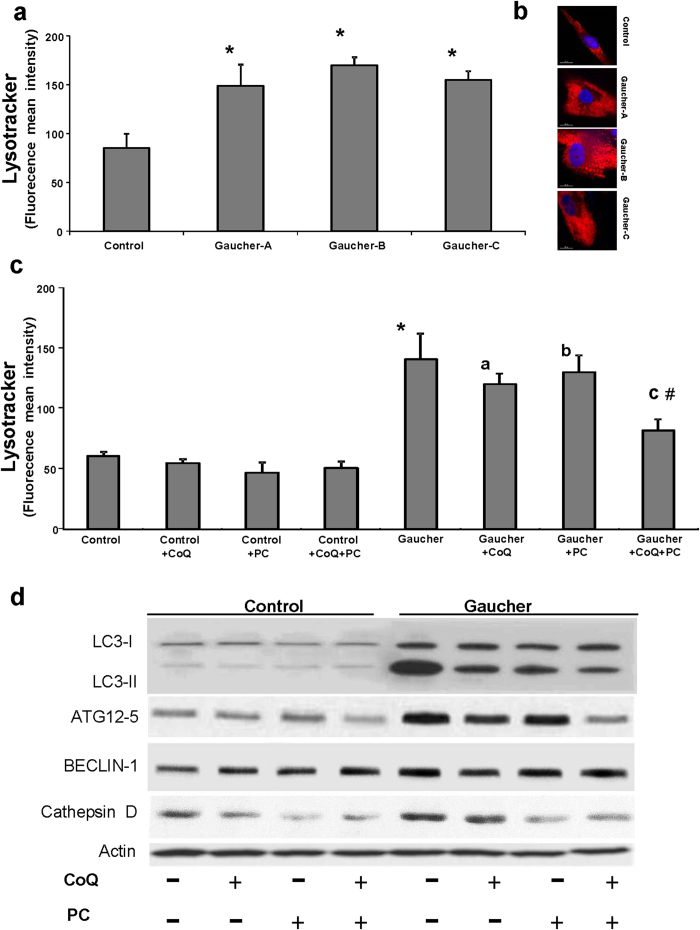
Increased expression of autophagic markers in Gaucher fibroblasts. **(a)** Quantification of acidic vacuoles in control and Gaucher fibroblasts by LysoTracker staining and flow cytometry analysis. **(b)** Representative LysoTracker staining images in control and Gaucher fibroblasts. Scale bar= 15 μm. **(c)** Effect of CoQ, PC and CoQ + PC on the amount of acidic vesicules in Gaucher-B fibroblasts. Control and Gaucher fibroblasts were cultured in the presence or absence of CoQ (25 μM), PC NAdBT-AIJ (25 μM) or CoQ + PC (25 μM + 25 μM) for 96 h. Acidic vacuoles were quantified by LysoTracker staining and flow cytometry analysis. For control cells, the data are the mean ± SD for experiments conducted on 2 different control cell lines. Data represent the mean ± SD of 3 separate experiments. *p < 0.01 between control and Gaucher fibroblasts. ^a^p < 0.05 between the presence and the absence of CoQ. ^b^p < 0.05 between the presence and the absence of PC. ^c^p < 0.05 between the presence and the absence of CoQ + PC. ^#^p < 0.05 between CoQ + PC and CoQ or PC treatment. **(d)** The expression levels of LC3-I (upper band) and LC3-II (lower band), ATG12, BECLIN1 and cathepsin D were determined in the control and Gaucher-B fibroblast cultures by Western blotting. The ATG12 band represents the Atg12-Atg5 conjugated form. Fibroblast cultures were grown in normal culture medium or in medium supplemented with CoQ (25 μM), PC (25 μM) or the combination of both CoQ + PC (25 μM + 25 μM) for 96 h. Actin was used as a loading control. The densitometric analysis of Western blottings is showed in Supplementary Figure S6.

Recent evidence suggests the involvement of ROS in autophagy activation^28^. To determine if autophagy was increased in Gaucher L444P fibroblasts, we first measured the amount of acidic vacuoles by LysoTracker staining and fluorescence microscopy and flow cytometry analysis. Using this technique, a 1.4 fold increase in LysoTracker staining was observed in Gaucher as compared to control fibroblasts (Fig. 4a,b). To elucidate whether autophagy in Gaucher fibroblasts could be attenuated by improving mitochondrial function by CoQ and mutant GCase folding by NAdBT-AIJ chaperone supplementation, we quantified the number of lysosomes in the various cultures following CoQ (25 μM), PC NAdBT-AIJ (25μM) or CoQ + PC supplementation. Treatment was associated with a reduction in the intensity of LysoTracker staining in Gaucher fibroblasts (Fig. 4c), indicating that lysosomal activity was reduced following CoQ or PC supplementation and more significantly with the combined treatment CoQ + PC. CoQ + PC effectiveness in reducing the amount of acidic vacuoles was confirmed in the three Gaucher cell lines (Supplementary Figure S5a and S5b).

To further verify that autophagy was activated in Gaucher fibroblasts, we examined the expression levels of autophagic and lysosomal proteins by Western blotting. First, we investigated the conversion of LC3-I (microtubule-associated light chain3) to LC3-II, which is closely correlated with the number of autophagosomes. The ratio of LC3-II to LC3-I was significantly increased in Gaucher fibroblasts (Fig. 4d), indicating enhanced autophagosome formation in Gaucher fibroblasts. As autophagosome formation involves an ubiquitin-like conjugation system in which Atg12 is covalently bound to Atg5, we also determined the expression levels of ATG12-ATG5 conjugates. ATG12-ATG5 expression levels were significantly increased in Gaucher fibroblasts. Likewise, the expression levels of BECLIN1, another essential protein required for the initiation of the formation of the autophagosome, was slightly increased in Gaucher fibroblasts when compared to control cells (Fig. 4d). We also studied autophagy activation in Gaucher fibroblasts by examining the expression levels of cathepsin D, a lysosomal enzyme. Compared to control cultures, there was a significant increase in cathepsin D expression levels in Gaucher fibroblasts. Supplementation with CoQ (25 μM) or PC NAdBT-AIJ (25 μM) partially reduced the expression levels of autophagic and lysosomal proteins (Fig. 4d). However, the combined treatment of both CoQ + PC was highly effective and restored the autophagic and lysosomal protein expression levels to near control levels (Fig. 4d). The densitometric analysis of Western blottings is showed in Supplementary Figure S6.

### Mitophagy in Gaucher Fibroblasts

**Figure 5:**
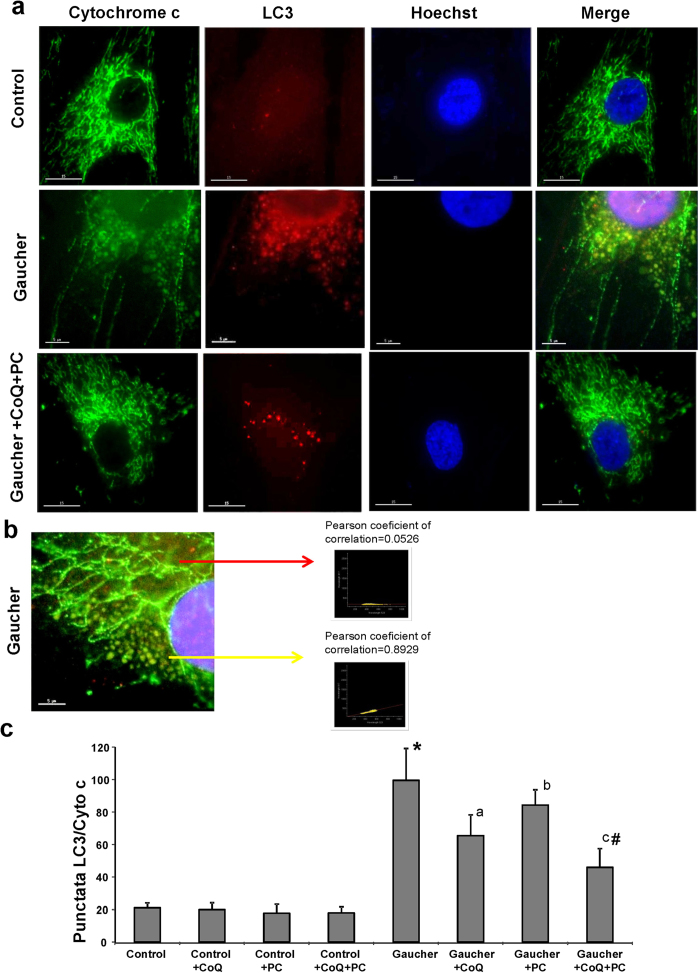
Autophagosome and mitochondria markers colocalization in Gaucher fibroblasts. **(a)** Image analysis of LC3 and cytochrome c immunostaining in control and Gaucher fibroblasts. Control and Gaucher-B fibroblasts were cultured in the presence or absence of CoQ (25 μM), PC NAdBT-AIJ (25 μM) or CoQ + PC (25 μM + 25 μM) for 96 h. Cells were fixed and immunostained with anti-LC3 (autophagosome marker) and cytochrome c (mitochondrial marker) and examined by fluorescence microscopy. Scale bars=15 (upper and lower pannel) or 5 μm (middle pannel). **(b)** Magnification of a small area in a Gaucher fibroblast. Yellow arrow shows autophagosomes with LC3 and cytochrome c colocalization. Red arrow shows tubular mitochondria without colocalization with LC3. Colocalization of both markers was assessed using the DeltaVision software and calculating the Pearson´s coefficient of correlation. Scale bar = 5 μm. **(c)** Quantification of LC3/cytochrome c puntacta in control and Gaucher fibroblasts incubated with or without CoQ, PC and CoQ+PC (n = 100 cells).*p < 0.01 between control and Gaucher fibroblasts. ^a^p < 0.05 between the presence and the absence of CoQ. ^b^p < 0.05 between the presence and the absence of PC. ^c^p < 0.05 between the presence and the absence of CoQ + PC. ^#^p < 0.05 between CoQ + PC and CoQ or PC treatment.

**Figure 6:**
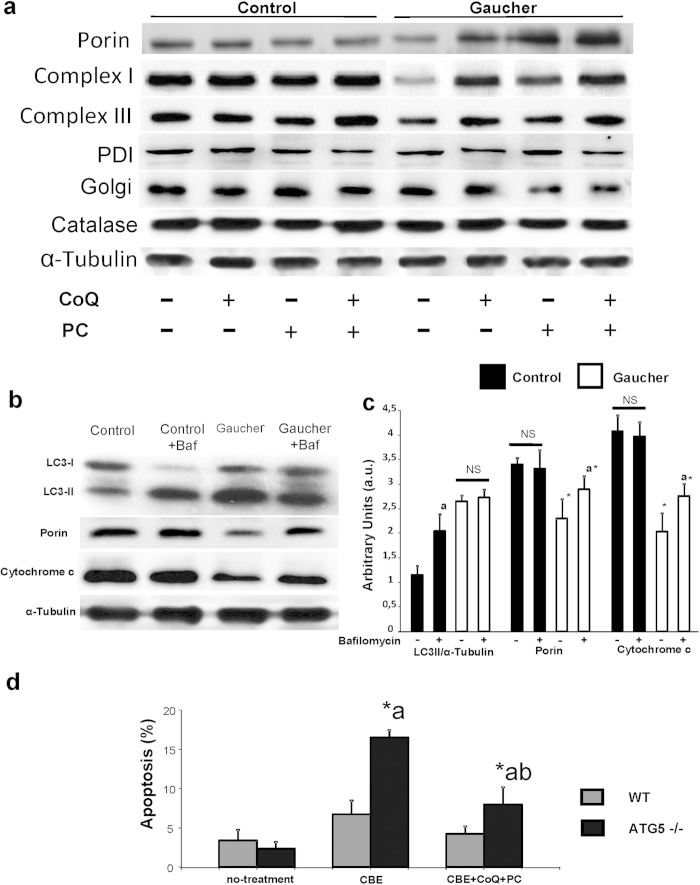
Mitophagy and impaired autophagic flux in Gaucher fibroblasts. **(a)** Western blot analysis of mitochondrial (complex I, 30 kDa subunit; complex II, 30 kDa subunit; complex III, core 1 subunit; complex IV, COX II subunit; and porin), Golgi (Golgi marker), endoplasmic reticulum (PDI), and peroxisome (catalase) proteins in control and Gaucher-B fibroblasts treated with CoQ (25 μM), PC NAdBT-AIJ (25 μM) or CoQ + PC (25 μM+25 μM) for 96 h. Alpha-tubulin was used as loading control. **(b)** Autophagy flux. Determination of LC3-II, porin and cytochrome c levels in the presence and absence of bafilomycin A1 in control and Gaucher-B fibroblasts. Control and Gaucher fibroblasts were incubated with bafilomycin A1 (100 nM for 12 h). Total cellular extracts were analyzed by immunoblotting with antibodies against LC3, porin and cytochrome c. α-Tubulin was used as a loading control. **(c)** Densitometry of Western blotting was performed using the ImageJ software. Data represent the mean ± SD of three separate experiments. *p < 0.01 between control and Gaucher fibroblasts. ^a^p < 0.05 between the presence and the absence of bafilomycin A1. **(d)** Apoptosis is increased in GCase-deficient Atg5-/- cells. Apoptosis is increased in GCase-deficient Atg5-/- cells. GCase deficiency in wild-type and Atg5-/- MEFs was induced by treatment with CBE 2,5 mM for 48 hours. Apoptosis was assessed in both autophagy proficient (wild-type), and autophagy-deficient cells (Atg5-/-) treated with CBE as described in Material and Methods. CBE-treated MEFs were also supplemented with, CoQ+PC NAdBT-AIJ for 96 hours to verify the specificity of apoptosis induction by GCase deficiency. Results are expressed as mean ± SD of three independent experiments *p < 0.01 between Atg5-/- and wild-type MEFs. ^a^p < 0.01 between the presence and the absence of CBE . ^b^p < 0.01 between Atg5-/- in the presence or absence of CoQ+PC.

To determine whether selective mitochondrial degradation or mitophagy was increased in Gaucher fibroblasts, we performed immunofluorescence double staining with antibodies against LC3 (autophagosome marker) and cytochrome c (mitochondrial marker) (Fig. 5a,b,c). LC3 staining was hardly detectable in control cells (Fig. 6a), whereas normal tubular mitochondria, negative for LC3, along with many small, fragmented mitochondria, positive for LC3, were observed in Gaucher fibroblasts (Fig. 5a,b,c). The population of small, rounded mitochondria showed a high colocalization of cytochrome c with LC3 staining, (r = 0.8929), whereas the tubular mitochondria did not (r = 0.0526) (Fig. 5a,b,c).

Supplementation with CoQ (25 μM) or PC NAdBT-AIJ (25 μM) partially reduced the number of LC3/cytochrome c punctata (Fig. 5c). However, the combined treatment of both CoQ+PC, was highly effective and drastically reduced the number of LC3/cytochrome c punctata (Fig. 5c).

Mitophagy activation in Gaucher fibroblasts was also confirmed by checking that the expression levels of mitochondrial proteins such as complex I (30 kDa subunit), complex III (core 1 subunit), and porin were reduced compared to controls fibroblasts and, on the contrary, the expression levels of Golgi (Golgi marker), endoplasmic reticulum (PDI, protein disulfide isomerase), and peroxisome (catalase) proteins markers were not affected (Fig. 6a). Supplementation with CoQ (25 μM) or PC NAdBT-AIJ (25 μM) partially restored mitochondrial protein expression levels (Fig. 6a). However, the combined treatment of both CoQ + PC was highly effective and mitochondrial protein expression levels were increased near to control values (Fig. 6a). The densitometric analysis of Western blottings is showed in Supplementary Figure S7.

### Autophagic Flux is Impaired in Gaucher Fibroblasts

To examine autophagosome/autophagolysosomal maturation and to ascertain whether or not the autophagic flux is impaired in Gaucher fibroblasts, we also checked the levels of LC3-II in the presence of bafilomycin A1 (Baf), a specific inhibitor of vacuolar H^+^-ATPases and a blocker of autophagosome-lysosome fusion. As expected, bafilomycin A1 treatment in control fibroblast cells led to a significant increase in the amount of LC3-II, suggesting that autophagic flux was normal (Fig. 6b,c). However, bafilomycin A1 treatment in Gaucher fibroblasts had no effect on LC3-II expression levels (Fig. 6b,c), indicating that autophagic flux was impaired in this case. We also examined whether mitochondrial proteins levels were affected by inhibition of autophagic flux. Inhibition of autophagosome-lysosome fusion by bafilomycin A1 induced an increase of porin and cytochrome c levels in Gaucher fibroblasts but not in control fibroblasts (Fig. 6a,b), suggesting increased mitochondrial delivery and degradation within autophagolysosomes in mutant cells.

Altogether, these results support the hypothesis that both mitophagy activation and impaired autophagic flux coexist in Gaucher fibroblasts.

### Autophagy is a Protective Mechanism in Gaucher Fibroblasts

To verify the role of autophagy in survival of GCase deficient cells, apoptosis was examined in GCase deficient wild-type and Atg5-/- mouse embryonic fibroblasts (MEFs). GCase deficiency in MEFs was produced by treatment with the specific GCase inhibitor CBE. We verified that CBE treatment indeed induced GCase deficiency in wild-type and Atg5-/- knockout fibroblasts (data not shown). We then analyzed cell viability and apoptosis in both GCase-deficient wild-type and knockout fibroblasts. GCase deficiency induced a high viability and low level of apoptosis in wild-type MEFs as determined by detecting caspase activation, cytochrome c release, and nuclear DNA condensation. In contrast, Atg5-/- MEFs were significantly more sensitive to GCase deficiency induction with a significant decrease in cell viability and a 1.5 fold increase in apoptosis respect to wild-type cells (Fig. 6d). CoQ+PC supplementation of CBE-treated MEFs reduced cell death to control values, confirming the specificity of apoptotic induction by GCase deficiency (Fig. 6d).

### Treatment with CoQ+PC NAdBT-AIJ Increases GCase Activity, Improves Enzyme Traffic and Reduces Intracellular GlcCer Levels

**Figure 7:**
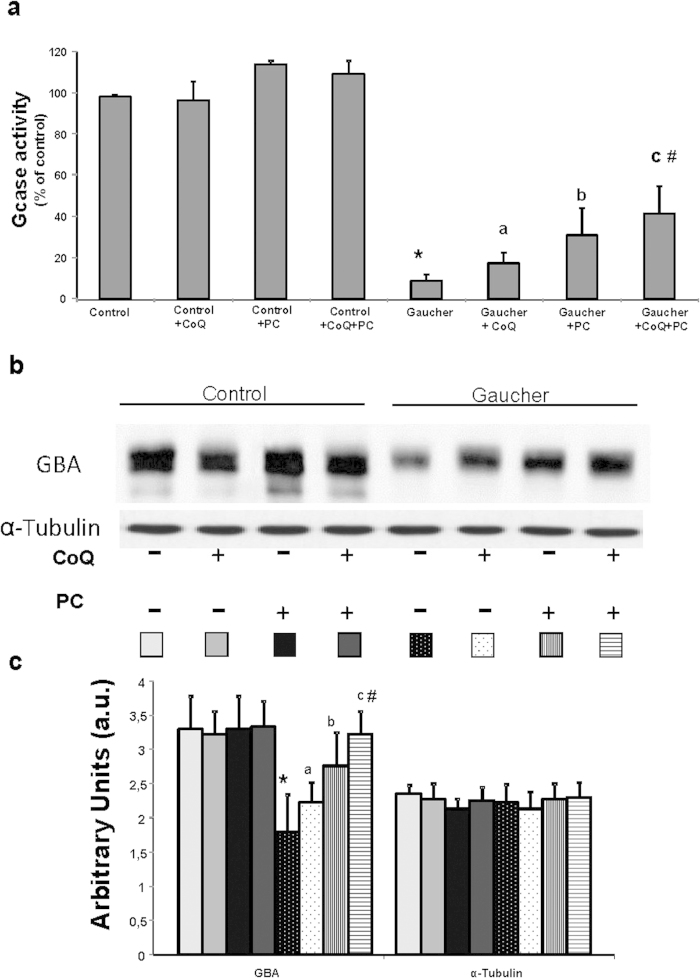
Treatment of Gaucher fibroblasts with CoQ and PC NAdBT-AIJ increases GCase activity. **(a)** GCase activities in control and Gaucher-B fibroblasts cultured in the absence or presence of CoQ (25 μM), PC NAdBT-AIJ (25 μM) and CoQ + PC (25 μM + 25 μM) for 96 h. GCase activities increase after CoQ, PC and markedly after CoQ+PC supplementation. Data, expressed as Gcase activity (% of control), represent the mean ± SD of 3 separate experiments. **(b)** GCase expression levels determined in control and Gaucher-B fibroblasts by Western blotting. Control and Gaucher fibroblast cultures were grown in normal culture medium or in medium supplemented with CoQ, PC and CoQ + PC for 96 h. Fibroblast protein extracts (50 μg) were separated on a 12.5% SDS-polyacrylamide gel and immunostained with an antibody against GCase. α-Tubulin was used as a loading control. **(c)** Densitometric analysis of Western blottings. Data represent the mean ± SD of 3 separate experiments. *p < 0.01 between control and Gaucher fibroblasts. ^a^p < 0.05 between the presence and the absence of CoQ. ^b^p < 0.05 between the presence and the absence of PC. ^c^p < 0.05 between the presence and the absence of CoQ + PC. ^#^p < 0.05 between CoQ + PC and CoQ or PC treatment.

GCase activities in the three Gaucher fibroblasts cell lines were reduced to 10%, 8% and 6% respectively, compared to control values (Supplementary Figure S8a). To elucidate whether GCase activities could be restored by improving mitochondrial function by CoQ and protein folding of the mutant GCase by PC NAdBT-AIJ supplementation, we determined GCase activities after treatment with CoQ (25 μM), PC NAdBT-AIJ (25 μM) or CoQ + PC. Supplementation with CoQ, PC and specially the combination of CoQ + PC induced a significant increase of 2-fold, 2.4-fold and 3.4-fold, respectively, of GCase activities (Fig. 7a). CoQ + PC treatment effectiveness in improving GCase activity was also confirmed in the three Gaucher cell lines (Supplementary Figure S8b).

Furthermore, deficient GCase activities were associated with reduced levels of GCase in Gaucher fibroblasts (Fig. 7b,c). As expected, both CoQ or PC and more significantly CoQ + PC treatment induced a marked increase in GCase expression levels (Fig. 7b,c).

**Figure 8:**
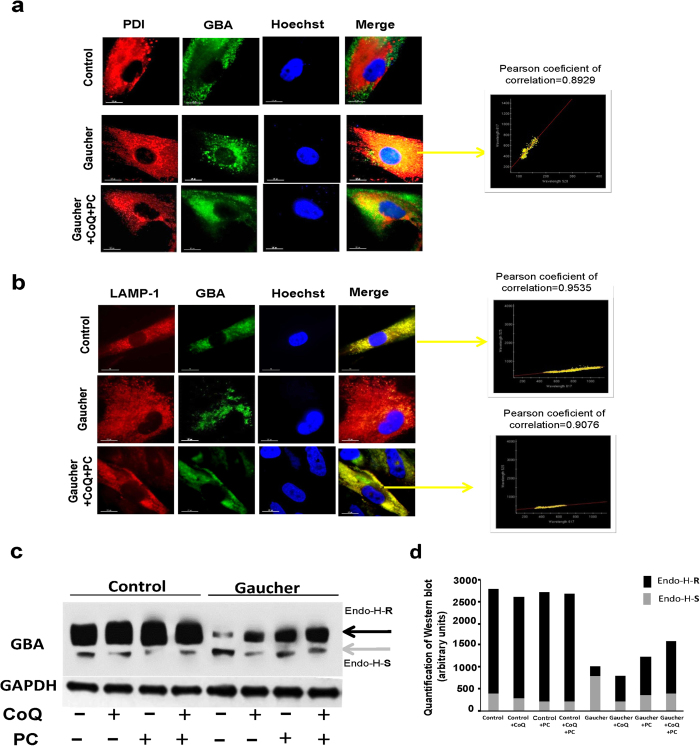
Treatment with CoQ and PC NAdBT-AIJ changes the intracellular localization of GCase in Gaucher fibroblasts. Effects of CoQ and PC NAdBT-AIJ on the traffic of GCase from the ER to lysosomes in Gaucher-B fibroblasts. Gaucher fibroblasts were treated with CoQ + PC (25 μM + 25 μM. **(a)** ER marker, PDI, or GCase are visualized as red or green, respectively. In the merged images, yellow denotes colocalization in the ER. Scale bar=15 μm. **(b)** Lysosomal marker, LAMP-1, or GCase are visualized as red or green, respectively. In the merged images, yellow denotes colocalization in lysosomes. Colocalization of both markers was assessed using the DeltaVision software and calculating the Pearson´s coefficient of correlation. Scale bar = 15 μm. **(c)** Representative Western blots of cell lysates treated with Endo-H. Cell lysates from control and Gaucher fibroblasts treated with CoQ, PC and CoQ + PC were subjected to Endo-H digestion and Western blot analysis with anti GCase antibody. Note the appearance, after Endo-H treatment, of a low molecular weight band in Gaucher disease samples (grey arrow), which represents endoplasmic reticulum retained GCase protein (Endo-H-**S**) and a high molecular weight band (Endo-H-**R**) which represents mature GCase (black arrow). GAPDH was used as a loading control. **(d)** Densitometric analysis of Western blotting. Quantification of Endo H assay using ImageJ analysis software. The gray portion of each bar represents Endo H-**S** bands and the black portion of the bar represents Endo H-**R** bands.

Accordingly, CoQ + PC treatment was also able to facilitate the efficient translocation of the mutant GCase to the lysosome compartment in Gaucher fibroblasts. The intracellular localization of mutant GCase before and after treatment was determined by using indirect fluorescent immunostaining. Cells were co-stained with IgGs against GCase and either a marker for lysosomes (LAMP-1) or an ER marker (PDI) (Fig. 8a,b). In untreated cells, GCase staining was diffuse and distinct from the punctate staining pattern of LAMP-1 (Fig. 8b). Instead, GCase staining colocalized (indicated by yellow colour) with the ER marker PDI (Fig. 8a). However, when Gaucher fibroblasts were treated with CoQ co-administered with PC NAdBT-AIJ, their GCase staining pattern increased in fluorescence intensity, became more punctate and exhibited a greater co-localization with LAMP-1, as indicated by the increased yellow colour in the Merge column (Fig. 8b). Furthermore, there was a notable decrease in the overlap between GCase and PDI staining of cells treated with CoQ + PC (Fig. 8a). In the case of CoQ + PC-treated cells, there was also an observable decrease in the overall intensity level of PDI staining, suggesting a decrease in ER stress, for which PDI is also a marker, as compared to untreated cells (Fig. 8a).

To verify increased mutant GCase folding and trafficking from the ER to the lysosomes, we performed endoglycosidase H (Endo-H) digestion assay on cell lysates from control and Gaucher fibroblasts, as it has been previously reported^15^. ER retained GCase proteins carrying N-linked glycans which are sensitive to Endo-H cleavage, producing a low molecular weight band of GCase on Western blotting (Endo-H-S).

In control fibroblasts most of GCase was Endo-H resistant (Endo-H-R), indicating that the protein passed already the mid-Golgi (probably mature lysosomal) (Fig. 8c,d). On the other, a lesser amount of the enzyme were Endo-H-R in Gaucher fibroblasts, suggesting that a significant fraction of GCase in these cells did not reach the mid-Golgi and therefore was not lysosomal. The increased levels of mutant GCase protein Endo-H-R in response to treatments, particularly CoQ + PC, support the hypothesis of a partial restoration of mutant GCase folding and post-ER trafficking.

Furthermore, to confirm the effectiveness of PC and/or CoQ treatment in improving Gcase activity, we quantified GlcCer levels by Dot-blot assays in control and Gaucher fibroblasts after CoQ (25 μM), PC NAdBT-AIJ (25 μM) or CoQ + PC (25 μM + 25 μM) supplementation. Both CoQ and PC and specially the combined treatment of CoQ+PC resulted in a significant reduction in GlcCer levels in Gaucher fibroblasts (Supplementary Figure S9a and S9b). Fluorescence microscopy examination also confirmed that CoQ + PC treatment reduced GlcCer accumulation both in the lysosomal and mitochondrial compartment (Supplementary Figure S10).

## Discussion

In this work, we studied the pathophysiology of the L444P/L444P mutation in primary cultured fibroblasts derived from GD patients with neurological involvement. We assessed both GCase deficiency and mitochondrial function in cultured fibroblasts. We found mitochondrial dysfunction associated with reduced activity and expression levels of GCase in Gaucher fibroblasts. In addition, mitophagy activation as a mechanism to eliminate dysfunctional mitochondria and impaired autophagic flux were observed in Gaucher fibroblasts. Next, we evaluated the effects of treatments targeting mitochondrial dysfunction and GCase misfolding on the improvement of the pathophysiological alterations of GD. Coenzyme Q_10_ (CoQ), a commercially available 1,4-benzoquinone derivative with cellular energy production and antioxidant functions occurring naturally in most eukaryotic cells, and *N*-[*N’*-(4-adamantan-1-ylcarboxamidobutyl)thiocarbamoyl]-1,6-anhydro-L-idonojirimycin (NAdBT-AIJ), a synthetic sp^2^-iminosugar-type PC recently shown to be active in restoring L444P GCase folding and trafficking in GD fibroblasts^20^, respectively, were used for those purposes. Our results showed that although single treatments are partially effective, the combined treatment of both CoQ and PC markedly increased GCase activity and enzyme trafficking to the lysosomes, and also improved mitochondrial function and the associated pathophysiological alterations.

Interestingly, the amount of CoQ, an essential electron carrier of the mitochondrial respiratory chain, was reduced in Gaucher fibroblasts. Concomitantly, a large proportion of the mitochondrial population was depolarized. Both alterations may further compromise normal mitochondrial function and cellular energy metabolism, which can aggravate the lysosomal functional defect. CoQ deficiency has been identified in a wide range of diseases, including mitochondrial diseases, lysosomal diseases, neurodegenerative diseases, such as Parkinson’s disease, cancer, fibromyalgia, and in patients undergoing statin treatment^29^.

In addition to CoQ deficiency and decreased mitochondrial membrane potential, we identified increased ROS production and H_2_O_2_ content, as well as increased expression of autophagic proteins. All these pathological conditions could be significantly ameliorated by CoQ + PC treatment in fibroblasts harboring the L444P/L444P mutation.

An alteration of autophagy in GD has been previously postulated^30^. GCase deficiency leads to both an induction of autophagy and an impairment of autophagic flux leading to accumulation of autophagosomes and autophagic substrates. The impairment in degradation of autophagic substrates may contribute to several aspects of Gaucher neuropathology, including the accumulation of ubiquitinated proteins and ROS production. Thus, oxidative stress may play a prominent role in the induction of the mitochondrial damage and autophagy activation, which we observed in Gaucher fibroblasts. In addition, the elimination of dysfunctional mitochondria could play a crucial role in protecting cells from the damage caused by perturbed mitochondrial function. Our finding that the autophagic protein LC3 colocalized with structurally abnormal mitochondria, but not with normal tubular mitochondria in Gaucher fibroblasts, suggests that autophagy specifically targets dysfunctional mitochondria in these cells. Formerly reported evidences on several LSDs showed increased ROS, dysfunctional mitochondria, blocked autophagy, aberrant inflammatory and apoptotic signaling and perturbed calcium homeostasis, among other biochemical alterations^1^.

Besides mitophagy activation, we found impaired autophagic flux in Gaucher fibroblasts. In GD, a reduction of autophagic flux may have a major impact on mitochondrial function and on cytoplasmic proteostasis. Constitutive macroautophagy maintains mitochondrial quality by selectively degrading dysfunctional mitochondria by mitophagy^31^. Mitochondrial proteins are consistently found in the proteomes of highly purified autolysosomes, especially subunits of the mitochondrial ATPase^32^. Reduced autophagic flux in GD may lead to the persistence of dysfunctional mitochondria, which is highly pronounced in other LSDs cells such as Batten’s disease neurons^33^. Several LSDs (mucolipidosis types IV, IIIA [pseudo-Hurler polydystrophy], and II [I-cell disease], late infantile neuronal ceroid lipifuscinosis [CLN2], mucopolysaccharidosis VI, and GM1 gangliosidosis) also display mitochondrial abnormalities, including replacement of the extended tubular mitochondrial network with high numbers of relatively rounded depolarized mitochondria^1^. Studies into aging and autophagosome formation have shown that mitochondria are involved in signaling pathways regulating apoptosis and innate immunity, and that reduced autophagic flux and subsequent accumulation of dysfunctional, reactive oxygen species–generating mitochondria renders cells more sensitive to apoptotic and inflammatory stimuli^34^. Therefore, the aberrant functioning of mitochondria may be responsible for apoptosis and inflammation in the CNS of multiple LSDs, including GD.

Defective activity of GCase in GD results in intracellular accumulation of the glycosphingolipids, glucosylceramide (GlcCer) and psychosine. Recently, it has been demonstrated in a mouse model of neuronopathic GD that glucosylceramide (GlcCer) and psychosine accumulation in GD brains precede the appearance of neuroinflammation, although only GlcCer accumulation correlated with neuroinflammation and neuron loss^35^. Some authors have hypothesized that variations in lipids involved in ceramide metabolism, including both GlcCer and ceramide, might play an important role in the associated mitochondrial dysfunction in GD. The possible deleterious effect of glycosphingolipids in the mitochondrial membrane and cellular bioenergetics was already discussed by Strasberg *et al*.^36^ and it has been suggested that the accumulation of the GlcCer and and GlcSph is the basis for the extensive neuronal cell loss in the neuronopathic forms GD type II/III^35^,^37^. Furthermore, it has been reported that the sphingolipid ceramides provoke oxidative stress by disrupting mitochondria and induce lethal mitophagy^38^. Interestingly, sub-cellular fractionation studies of Gaucher fibroblasts showed that GlcCer was not confined to the lysosomes but its concentration increases throughout the cell^39^. In agreement with these results, we found that GlcCer is accumulated mainly in the lysosomal and mitochondrial compartments in Gaucher fibroblasts. We propose that both accumulation of GlcCer and impairment of autophagic flux induce mitochondrial dysfunction in Gaucher fibroblast. Dysfunctional mitochondria are involved in the pathogenesis of several neurodegenerative diseases, but the experimental evidence for a role in altered mitochondria dynamics is especially strong in PD^6^. The proper elimination of damaged mitochondria is needed in post-mitotic neurons because progressive accumulation of damaged mitochondria might lead to eventual cell death.

Mitochondrial dysfunction with reduced respiratory chain complex activities, increased ROS production and decreased potential in neurons and astrocytes has recently been reported in a mouse model of type II neuronopathic Gaucher disease^37^. Thus, a primary lysosomal defect may cause accumulation of dysfunctional mitochondria. The neurological and pathological symptoms observed in Gaucher disease patients are similar to those displayed in Parkinson’s disease, including loss of dopaminergic neurons in the substantia nigra, alpha-synuclein accumulation, tremors at rest, bradykinesia, and rigidity^6^. Indeed, parkinsonism has been reported in many Gaucher disease patients, suggesting a mechanistic link between the two disease states. Further supporting this, large, multicenter genome-wide association studies have reported a high incidence of GBA mutations in patients with sporadic Parkinson’s disease and an increased risk of developing Parkinson’s disease in GBA carriers^40^.

An emerging strategy for the treatment of LSDs is pharmacological chaperone therapy (PCT), based on the use of chaperone molecules that assist the folding of mutated enzymes and improve their stability and lysosomal trafficking^41^. These PC can cross the BBB and therefore have a potential for the treatment of diseases with CNS involvement^42^. Our findings confirmed previous results showing the effectiveness of bicyclic derivatives of L-idonojirimycin as pharmacological chaperones for neuronopathic forms of Gaucher disease^20^. The PC compound NAdBT-AIJ increased GCase activity and also facilitated translocation of the enzyme to the lysosome in Gaucher fibroblasts harboring the L444P mutation.

Likewise, given that defects in energy metabolism and oxidative stress have been demonstrated to play a role in the pathogenesis of GD, we envisioned that the treatment with coenzyme CoQ could also exert beneficial therapeutic effects. The fundamental role of CoQ in mitochondrial bioenergetics and its well-acknowledged antioxidant properties constitute the basis for its clinical applications, although some of its effects may be related to a gene induction mechanism^29^. Interestingly, CoQ is also able to cross the BBB^43^. Therefore, the combined treatment of CoQ with PC NAdBT-AIJ may represent a therapeutic strategy, supplementary or alternative to ERT in Gaucher patients with CNS involvement. Indeed, our results suggest that the combined treatment of CoQ with PC NAdBT-AIJ is high effective in improving the biochemical phenotype in cellular models of GD harboring the L444P/L444P mutation.

In summary, our studies provide experimental evidence that targeting mitochondrial dysfunction and GCase misfolding by the combined supplementation of CoQ and the L444P-active pharmacological chaperone NAdBT-AIJ could be beneficial in the treatment of patients with neuronopathic GD. However, additional experiments in neuronal or animal models are essential for the confirmation of these results.

Our findings support the hypothesis that secondary mitochondrial dysfunction by GlcCer accumulation aggravates the effects of the L444P/L444P mutation and therefore, the improvement of mitochondrial function by CoQ treatment also improves the activity of the mutant GCase. The fact that both CoQ and PC NAdBT-AIJ can cross the BBB makes them promising candidates for the treatment of neuronopathic forms of GD that are not responsive to ERT.

## Material and Methods

### Reagents

Monoclonal Anti-Actin antibody, rabbit anti-VDAC1/Porin, rabbit anti-BECLIN1 and 4-methylumbelliferyl β-D-glucopyranoside substrate were all obtained from Sigma- Aldrich (St. Louis, MO). Monoclonal antibodies against complex III (core 1 subunit) and complex I (30 kDa subunit), Mitosox Red, CMH_2_-DCFDA, 10-N-nonyl acridine orange (NAO), MitoTracker, LysoTracker, tetramethylrhodamine methyl ester (TMRM), MitoTracker Green FM (MTG) and Hoechst 33342 were obtained from Invitrogen/Molecular Probes (Eugene, OR). Anti-GCase was obtained from Abcam. Anti-cytochrome c antibody was obtained from BD Biosciences Pharmingen (San Jose, CA) and anti-GAPDH (Glyceraldehyde 3-phosphate dehydrogenase) monoclonal antibody (clone 6 C5) was from Calbiochem-Merck Chemicals Ltd. (Nottingham, UK). Anti-hATG12 and anti-hATG5 were obtained from Biosensis (South Australia, Australia). Conduritol-B-epoxide (CBE), Anti-MAP LC3 (N-20), anti-catalase (H-300), anti-PDI (H-160), anti-Golgi marker (AE-6), anti-Cathepsin D and anti-LAMP-1 were obtained from Santa Cruz Biotechnology (Santa Cruz, CA). Protease inhibitors were obtained from Boehringer Mannheim (Indianapolis, IN). All other chemicals were purchased from Sigma-Aldrich. The anti-GlcCer rabbit anti-serum was purchased from Glycobiotech GmbH (Kükels, Germany). Glucocerebrosides from Gaucher’s spleen (GlcCer) was obtained from Matreya LCC (Pleasant Gap, PA, USA). Endoglycosidase-H was obtained from New England Biolabs (Hitchin, UK).

### Fibroblast Cultures

Cultured fibroblasts from 3 Gaucher patients (Gaucher-A, Gaucher-B and Gaucher-C) homozygous for L444P GCase were obtained from Coriell Cell Repositories (Camden, NJ, USA). Control primary fibroblasts were obtained from healthy volunteers; any control value represent the mean value for at least two control fibroblasts cell lines. Samples from patients and controls were obtained according to the Helsinki Declarations of 1964, as revised in 2001. This study has been approved by the institutional ethical committee of Pablo de Olavide University. Informed consent was obtained from all subjects biopsied. Control and Gaucher fibroblasts were cultured at 37 °C in Dulbecco’s Modified Eagle Medium (DMEM) (4.5 g/L glucose), plus pyruvate supplemented with 20% fetal bovine serum (FBS), and an antibiotic/anti-mycotic solution.

Mouse Embryonic Fibroblasts (MEFs) derived from wild-type and Atg5-/- mouse embryos were a kind gift of Noboru Mizushima, Tokyo Medical and Dental University, Japan^44^.

### Synthesis of the Pharmacological Chaperone NAdBT-AIJ

The preparation of the PC NAdBT-AIJ was accomplished in two steps and 95% overall yield from 5-amino-5-deoxy-1,2-O-isopropylidene-β-L-idofuranose, which at its turn is readily accessible from commercial D-glucuronolactone, by nucleophilic addition of 4-(1-adamantanylcarboxamido)butyl isothiocyanate^45^ followed by acid-catalyzed acetonide hydrolysis of the resulting thiourea adduct, following the procedure previously reported^20^.

### Treatment of Fibroblasts Cell Lines

Cells were also cultured with or without 25 μM CoQ and/or 25 μM PC NAdBT-AIJ for 96 h. CoQ and PC NAdBT-AIJ concentrations were selected based on the optimal concentration in increasing GCase activity in Gaucher fibroblasts.

### Mitochondrial Respiratory Chain and Citrate Synthase Enzyme Activities

Activities of Activities of NADH:coenzyme Q1 oxidoreductase (complex I), succinate dehydrogenase (complex II), ubiquinol:cytochrome c oxidoreductase (complex III), succinate:cytochrome c reductase (complex II+III) and citrate synthase (CS) were determined in sonicated permeabilized fibroblasts using previously described spectrophotometric methods^46^. Results are expressed as % of activity respect to control values. Proteins of fibroblasts homogenates were analyzed by the Lowry procedure^47^.

### Measurement of CoQ Levels

CoQ levels in cultured fibroblasts were performed using a method previously described by our group^48^.

### Adenosine-5’-Triphosphate Levels

Adenosine-5’-triphosphate (ATP) levels were determined using a bioluminescence assay (ATP determination kit, Invitrogen- Molecular Probes, Eugene, OR).

### Proliferation Rate

Two hundred thousand cells were cultured with or without 25 μM CoQ and/or 25 μM PC for 96 h. Cell counting was performed from 10 high power fields using an inverted microscope and a 40× objective.

### GCase Activity Assay

GCase activities in cell lysates were determined by using 4-methylumbelliferone-conjugated β-D-glucopyranoside as a substrate. The lysates (10 μL) were incubated at 37 °C with the substrate solution (20 μL) in 0.1 M citrate buffer, pH 5.2, supplemented with sodium taurocholate (0.8% w/v) for 1 h. The reactions were terminated by adding 0.2 μL of 0.2 M glycine sodium hydroxide buffer (pH 10.7). The liberated 4-methylumbelliferone was measured spectrophotometrically using a fluorescence Microplate Reader (excitation wave length: 340 nm; emission: 460 nm). One unit of enzyme activity was defined as 1 nmol of 4-methylumbelliferone released per hour and normalized for the amount of protein contained in the lysates.

### Immunofluorescence Microscopy

Immunofluorescence microscopy was performed using standard methods as previously described^49^. Cover slips were analyzed using a fluorescence microscope (Leica DMRE, Leica Microsystems GmbH, Wetzlar, Germany). Deconvolution studies and 3-dimensional projections were performed using a DeltaVision system (Applied Precision, Issaquah, WA) with an Olympus IX-71microscope. The deconvolved images were derived from optical sections taken at 30-nm intervals using a 60× PLAPON objective with a 1.42 numerical aperture.

### Measurement of Mitochondrial Reactive Oxygen Species (ROS) Generation

Mitochondrial ROS generation was assessed using the mitochondrial superoxide indicator MitoSOX Red, according to the manufacturer’s instructions. ROS levels were expressed relative to mitochondrial mass (ROS signal/NAO signal), determined by flow cytometry and fluorescence microscopy of cells stained with 10 μM NAO for 10 minutes at 37 °C in the dark.

### Measurement of Intracellular H_2_O_2_ Content

H_2_O_2_ levels were measured using non fluorescent CMH_2_-DCFDA (5-[and-6]-chloromethyl-2’,7’-dichlorodihydrofluoresceindiacetate, acetyl ester), which diffuses across membranes and is oxidized to fluorescent dichlorofluorescein (DCF). Cultured cells were rinsed in phosphate-buffered saline (PBS), incubated with CMH_2_-DCFDA diluted in medium at 5 μM for 30 minutes at 37 °C. After that, cells were washed, trypsinized, and re-suspended in pre-warmed PBS at 37 °C. Cells were then analyzed by flow cytometry.

### Determination of Mitochondrial Membrane Potential (ΔΨm)

Cells were cultured in six-well 35 mm plates and, at confluence, were incubated for 30 minutes with 100 nM Mitotracker Red CMXRos. Cells washed with fresh medium were subsequently harvested and analyzed by flow cytometry. Fibroblasts were grown on 1-mm width (Goldseal No. 1, Thermo Fisher Scientific Inc, Waltham, MA) glass coverslips for 24 to 48 hours, incubated with 100 nM MitoTracker Red for 30 minutes, fixed, and immunostained with anti-cytochrome c (a mitochondrial marker), and examined by fluorescence microscopy. Colocalization of both markers and quantification of MitoTracker fluorescence in individual mitochondria was assessed by the DeltaVision software (Applied Precision).

Mitochondria were classified as small-depolarized and tubular-polarized mitochondria (tubular, mitochondrial tubule of 5 μm in length is present in a cell). When this network was disrupted and mitochondria appeared predominantly spherical and small they were classified as depolarized. Quantification of fluorescence signal was performed in 100 cells using the ImageJ software (National Institutes of Health, Bethesda, Maryland, USA).

ΔΨm was also measured by double staining with 20 nM TMRM (a potentiometric fluorescent indicator) and 100 nM MTG (a non-potentiometric dyes that relies on mitochondrial mass independent of ΔΨm) and flow cytometry analysis. Relative ΔΨm values were calculated by determining the ratio of TMRM signal to MTG fluorescence in arbitrary units.

### Immunoblotting Analysis

Western blotting was performed using a standard protocol^48^ and the Immun Star HRP detection kit (Bio-Rad Laboratories Inc., Hercules, CA, USA).

### LysoTracker Red assay

LysoTracker Red (100 nM) was added to cultured fibroblasts for 30 minutes each, well was washed twice with fresh DMEM, and then the cells were fixed with 2% paraformaldehyde in PBS for 10 minutes at 4 °C. LysoTracker fluorescence was quantified by flow cytometry.

### Analysis of apoptotic and viable cells

Apoptosis was assessed by observing nuclei fragmentation by Hoechst staining, cytochrome *c* release, and caspase 3 activation. Viable cells were determined from their normal nuclear morphology and exclusion of propidium iodide. In each case 10 random fields and more of 500 cells were counted.

### Dot blot assay for glucosylceramide (GlcCer)

Intracellular GlcCer levels was performed by a dot blot assays as described previously^50^.

### Endoglycosidase-H treatment

Samples of cell lysates, containing 70 μg of protein, were denatured with glycoprotein denaturing buffer (New England Biolabs 5% SDS, 0.4 M dithiothreitol) at 100 °C for 15 minutes. The lysate was subjected to an overnight incubation with endoglycosidase-H and reaction buffer (New England Biolabs 0.5 M sodium citrate, pH 5.5) according to the manufacturer’s instructions. The lysates were then analyzed by Western blot with the anti GCase antibody.

### Statistical Analysis

All results are expressed as mean±SD of 3 independent experiments. The measurements were statistically analyzed using the Student’s *t* test for comparing 2 groups and analysis of variance for more than 2 groups. The level of significance was set at p < 0.05.

## Additional Information

**How to cite this article**: de la Mata, M. *et al.* Pharmacological Chaperones and Coenzyme Q_10_ Treatment Improves Mutant ß-Glucocerebrosidase Activity and Mitochondrial Function in Neuronopathic Forms of Gaucher Disease. *Sci. Rep.* **5**, 10903; doi: 10.1038/srep10903 (2015).

## Supplementary Material

Supplementary Information
