# Supplementary material for: Pharmacological Chaperones and Coenzyme Q10 Treatment Improves Mutant β-Glucocerebrosidase Activity and Mitochondrial Function in Neuronopathic Forms of Gaucher Disease
Source: Sci Rep. 2015 Jun 5;5:10903. doi: 10.1038/srep10903 (PMC4456666; doi:10.1038/srep10903)
Supplement: Supplementary Information [file srep10903-s1.pdf]

Pharmacological Chaperones and Coenzyme Q_{10} Treatment Improves Mutant β-Glucocerebrosidase Activity and Mitochondrial Function in Neuronopathic Forms of Gaucher Disease

Mario de la Mata[1], David Cotán[1], Manuel Oropesa-Ávila[1], Juan Garrido-Maraver[1], Mario D. Cordero[2], Marina Villanueva Paz[1], Ana Delgado Pavón[1], Elizabet Alcocer-Gómez[1], Isabel de Lavera[1], Patricia Ybot-González[3], Ana Paula Zaderenko[4], Carmen Ortiz Mellet[5], José M. García Fernández[6], José A. Sánchez-Alcázar[1]*.

[1]Centro Andaluz de Biología del Desarrollo (CABD-CSIC-Universidad Pablo de Olavide), and Centro de Investigación Biomédica en Red: Enfermedades Raras, Instituto de Salud Carlos III, Sevilla. [2]Facultad de Odontología, Universidad de Sevilla, Sevilla. [3]Instituto de Biomedicina de Sevilla (IBIS)-CSIC, Hospital Virgen del Rocío, Sevilla 41013. [4]Sistemas Físicos, Químicos y Naturales-Universidad Pablo de Olavide. [5]Dept. Química Orgánica, Facultad de Química, Universidad de Sevilla, Sevilla. [6]Instituto de Investigaciones Químicas (IIQ) CSIC-Universidad de Sevilla, Sevilla, Spain.

*Author for correspondence:

José A. Sánchez Alcázar. Centro Andaluz de Biología del Desarrollo (CABD). Consejo Superior de Investigaciones Científicas. Universidad Pablo de Olavide. Carretera de Utrera Km 1, Sevilla 41013, Spain.

Phone: 34 954978071.   FAX: 34 954349376.

Email: jasanalc@upo.es; Web page: http://www.upo.es/CABD/

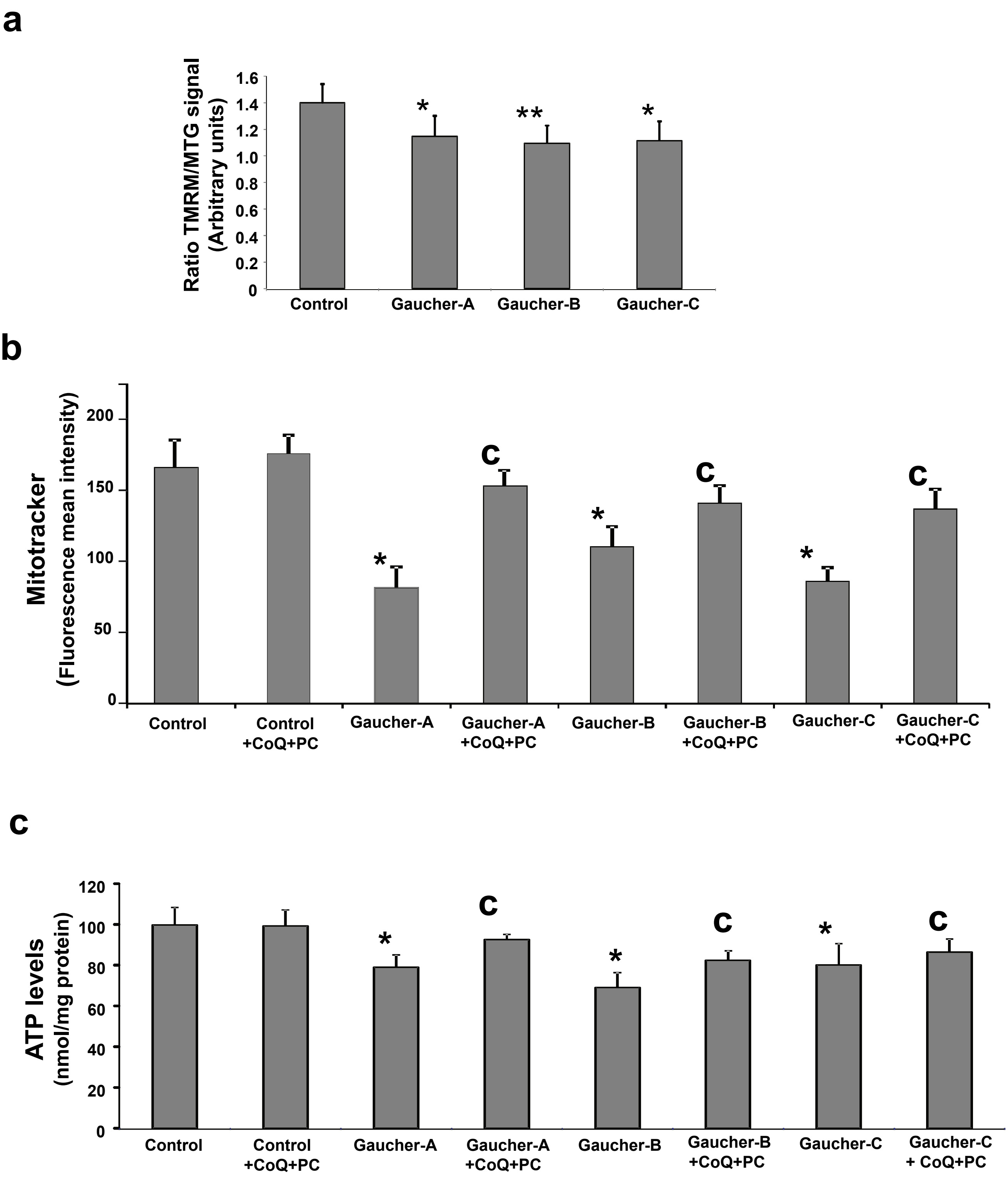

Figure S1. Mitochondrial function in Gaucher fibroblasts is recovered by CoQ and PC NAdBT-AIJ treatments. **(a)** Mitochondrial membrane potential ($\Delta\Psi m$) in control and Gaucher fibroblasts was assessed by flow cytometry using the ratio TMRM/MTG signal. **(b)** $\Delta\Psi m$ was assessed by flow cytometry using MitoTracker Red staining. (c) Adenosine-5'-triphosphate (ATP) levels in control and Gaucher fibroblasts. Data represent the mean±SD of 3 separate experiments. *$p<0.01$ between control and Gaucher fibroblasts. $^{c}p<0.05$ between the presence and the absence of CoQ+PC.

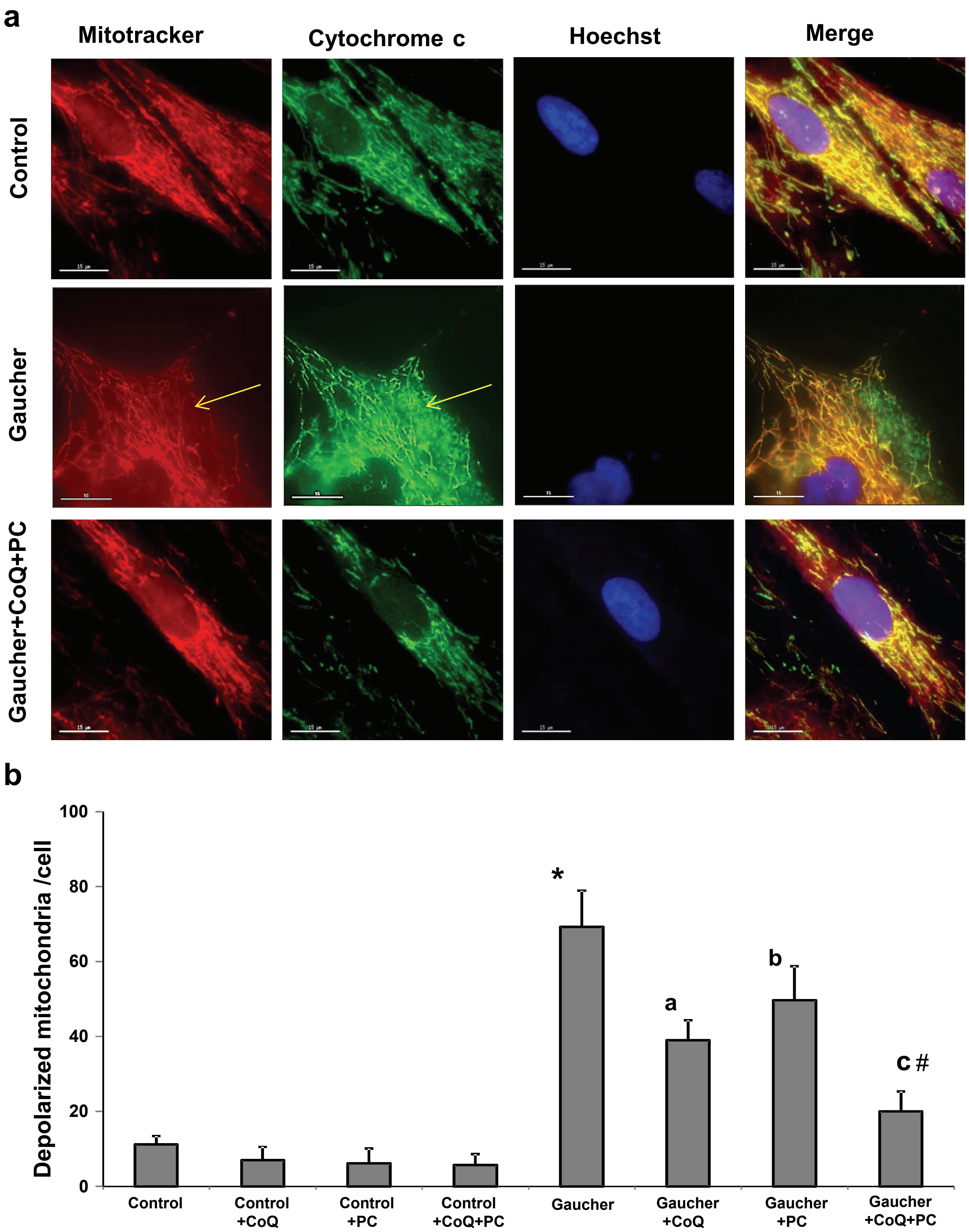

Figure S2. CoQ and PC NAdBT-AIJ treatments restore tubular mitochondrial network in Gaucher fibroblasts. **(a)** Representative images of MitoTracker and cytochrome c staining. Yellow arrows indicate small depolarized mitochondria. **(b)** Quantification of depolarized mitochondria by fluorescence imaging analysis. Data represent the mean±SD of 3 separate experiments. *$p<0.01$ between control and Gaucher fibroblasts. $^{a}p<0.05$ between the presence and the absence of CoQ. $^{b}p<0.05$ between the presence and the absence of PC. $^{c}p<0.05$ between the presence and the absence of CoQ+PC. #$p<0.05$ between CoQ+PC and CoQ or PC treatment.

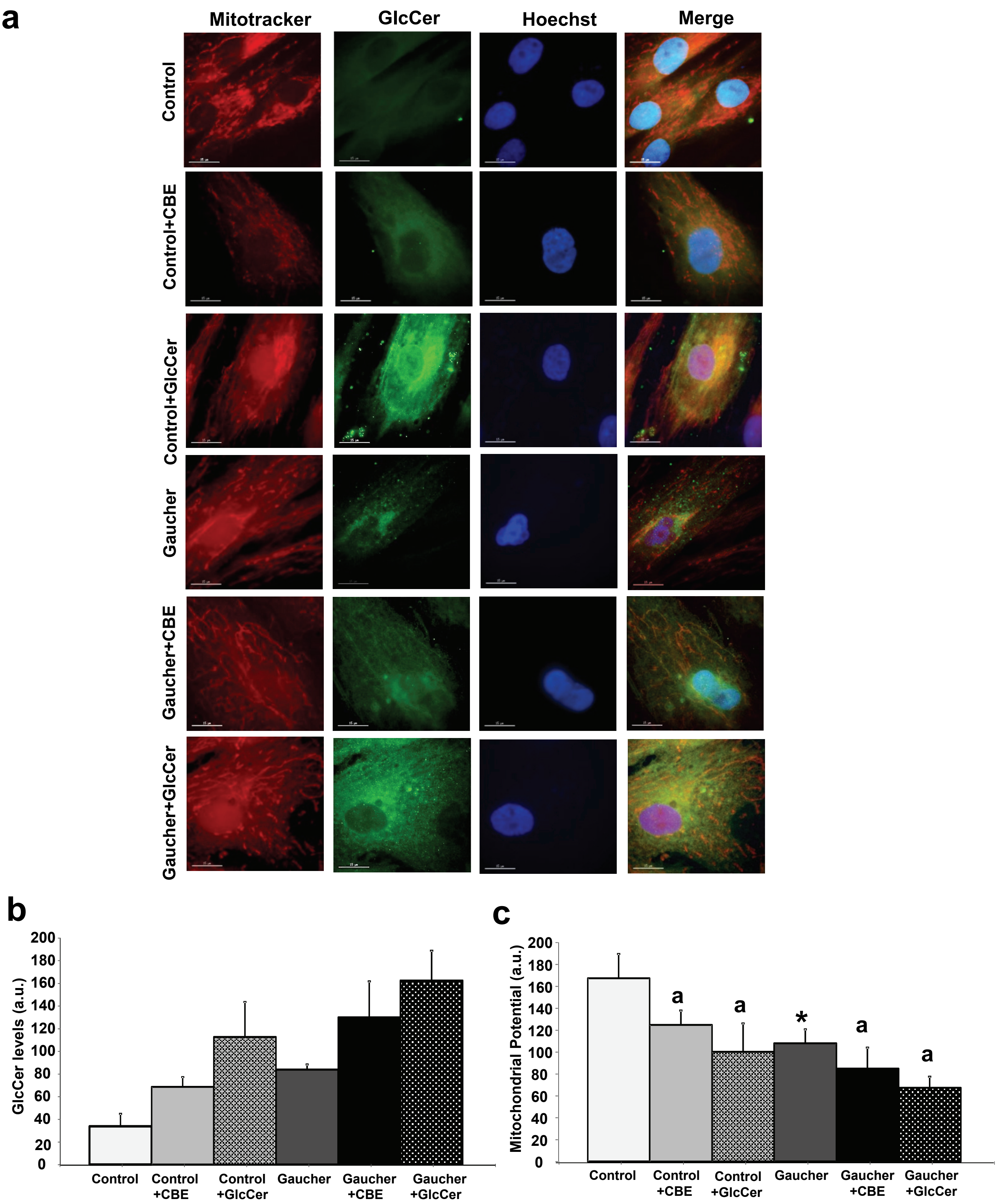

Figure S3. Effect of GlcCer accumulation on mitochondrial function. **(a)** Representative images of Mitotracker and anti-GlcCer anti-sera staining in control and Gaucher fibroblasts. **(b)** Quantification of levels of GlcCer by fluorescence imaging analysis. (c) Quantification of ΔΨm in control and Gaucher fibroblasts. Data represent the mean±SD of 3 separate experiments. *p<0.01 between control and Gaucher fibroblasts. [a]p<0.01 between untreated and treated with CBE or GlcCer.

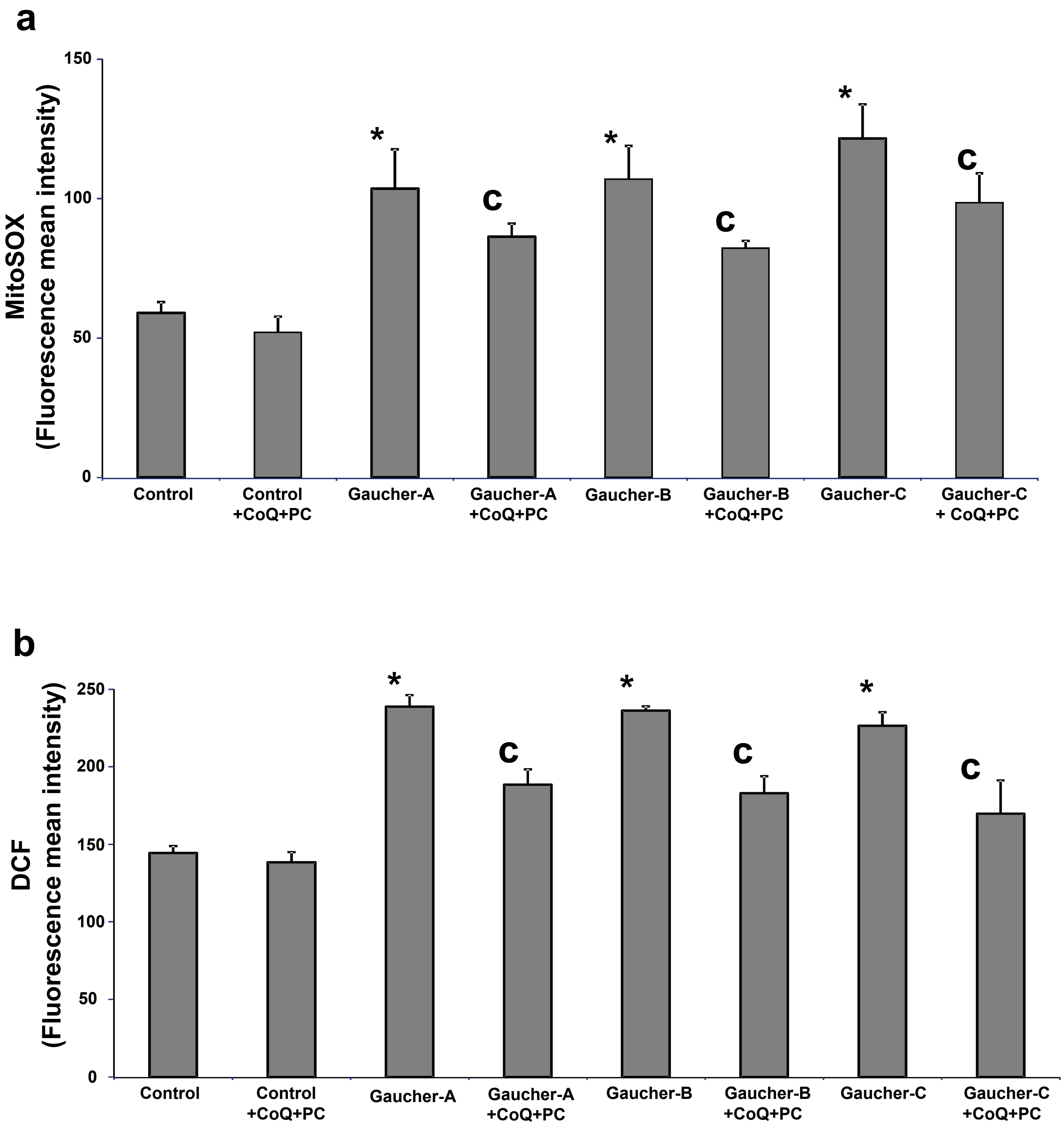

Figure S4. CoQ and PC NAdBT-AIJ treatments reduces oxidative stress in Gaucher fibroblasts. **(a)** Mitochondrial ROS levels in control and Gaucher fibroblasts. **(b)** H_2O_2 levels in control and Gaucher fibroblasts. The mean±SD of 3 independent experiments are showed. *p<0.01 between control and Gaucher fibroblasts. ^{c}p<0.05 between the presence and the absence of CoQ+PC.

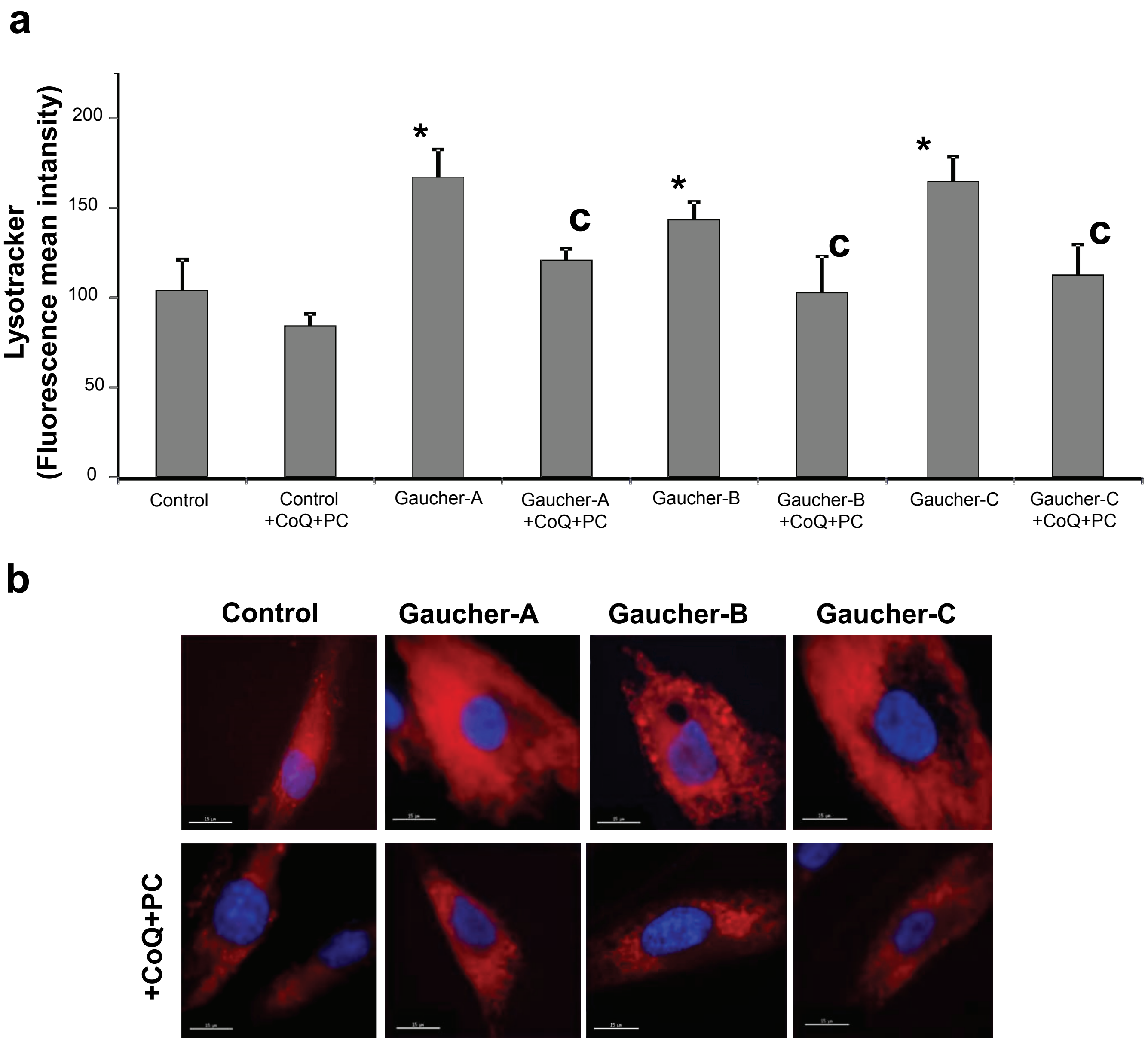

Figure S5. (a) Increased expression of autophagic markers in Gaucher fibroblasts. Effect of CoQ+PC NAdBT-AIJ treatment on the amount of acidic vesicules in Gaucher fibroblasts. For control cells, the data are the mean±SD for experiments conducted on 3 different control cell lines. Data represent the mean±SD of 3 separate experiments. *p<0.01 between control and Gaucher fibroblasts. ^{c}p<0.05 between the presence and the absence of CoQ+PC. **(b)** Representative LysotrackerTracker staining images in control and Gaucher fibroblasts after CoQ, PC or CoQ+PC treatments.

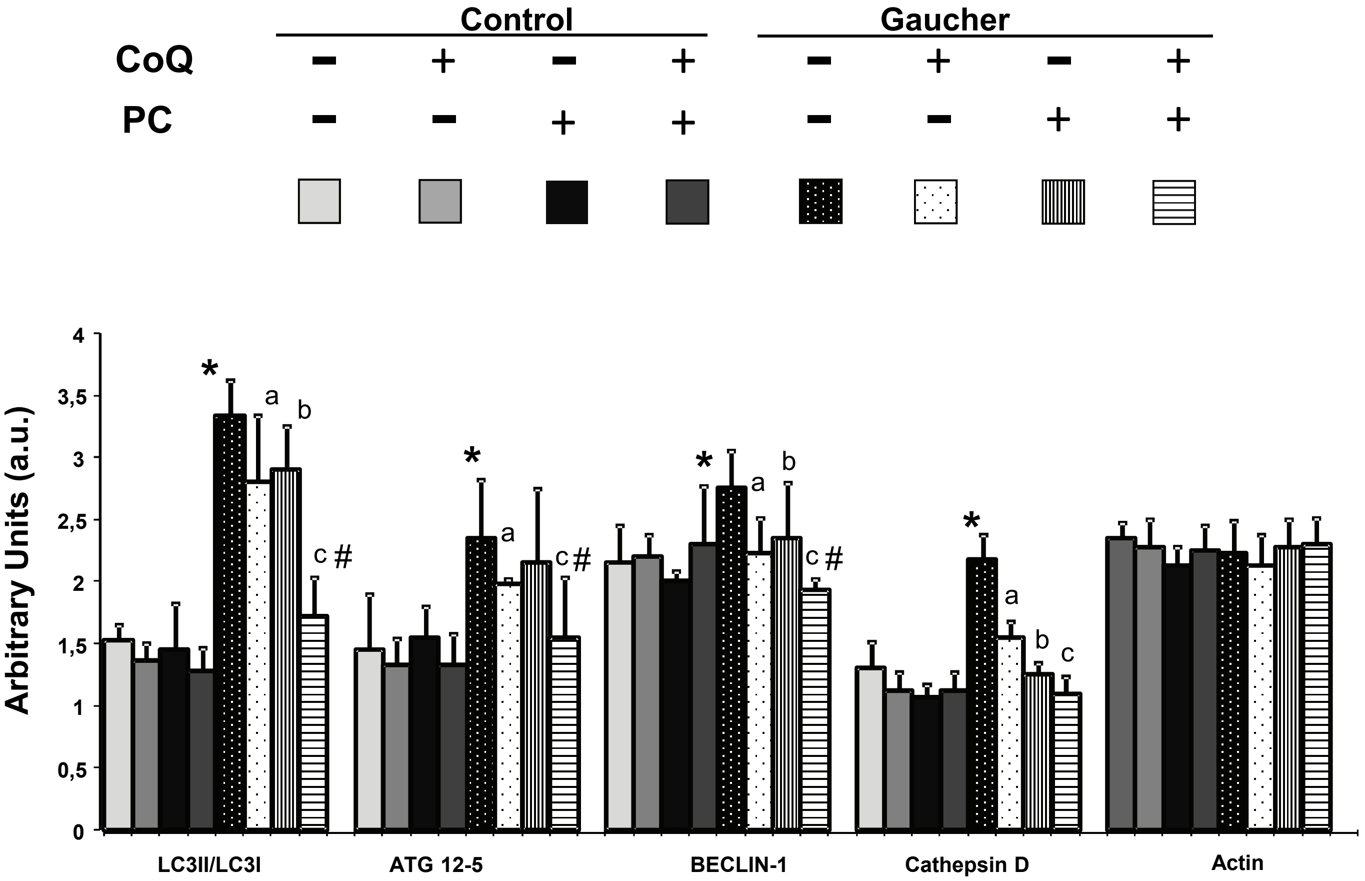

Figure S6. Densitometric analysis of Western blottings of Figure 4C. Data represent the mean±SD of 3 separate experiments. *$p<0.01$ between control and Gaucher fibroblasts. [a]$p<0.05$ between the presence and the absence of CoQ. [b]$p<0.05$ between the presence and the absence of PC. [c]$p<0.05$ between the presence and the absence of CoQ+PC. [#]$p<0.05$ between CoQ+PC and CoQ or PC treatment.

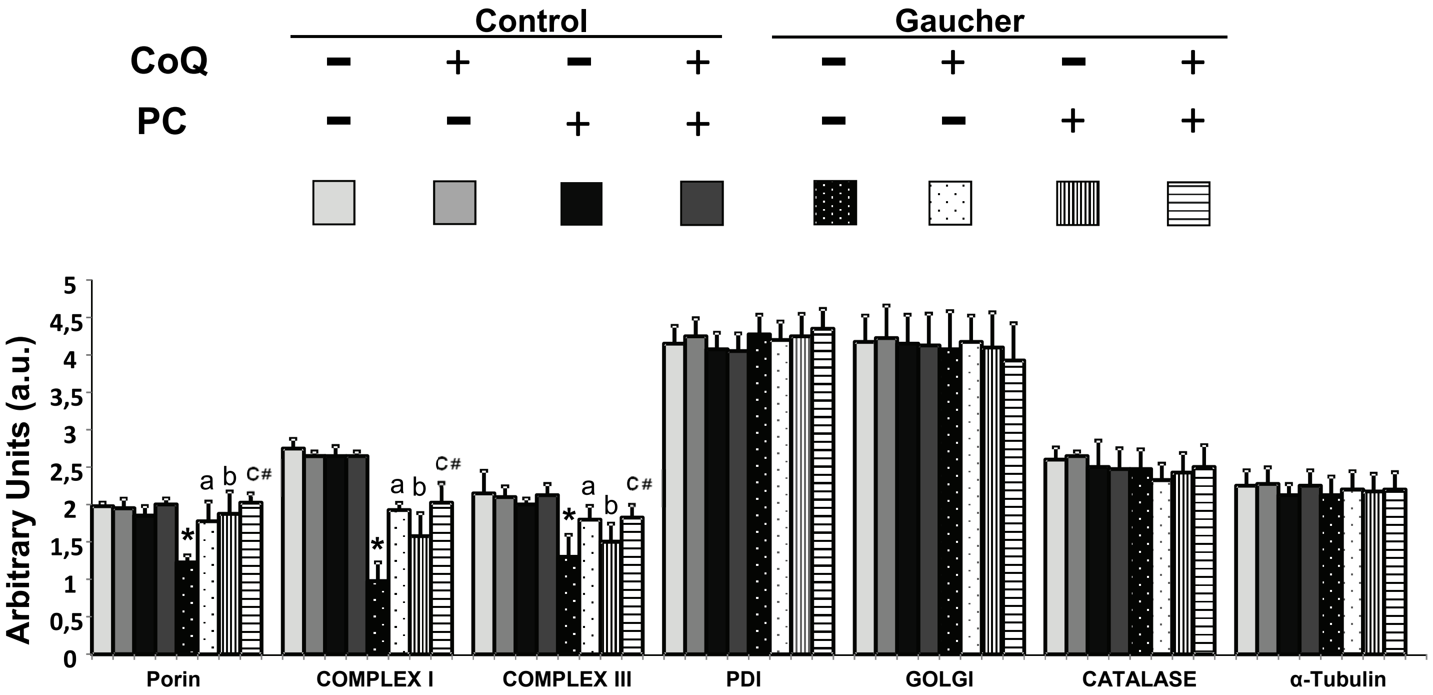

Figure S7. Densitometric analysis of Western blottings of Figure 6A. Data represent the mean±SD of 3 separate experiments. *p<0.01 between control and Gaucher fibroblasts. [a]p<0.05 between the presence and the absence of CoQ. [b]p<0.05 between the presence and the absence of PC. [c]p<0.05 between the presence and the absence of CoQ+PC. [#]p<0.05 between CoQ+PC and CoQ or PC treatment.

a

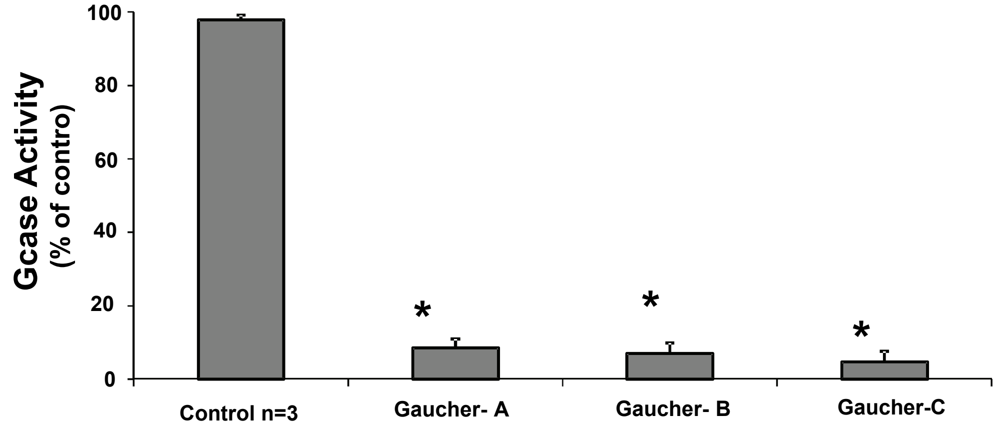

b

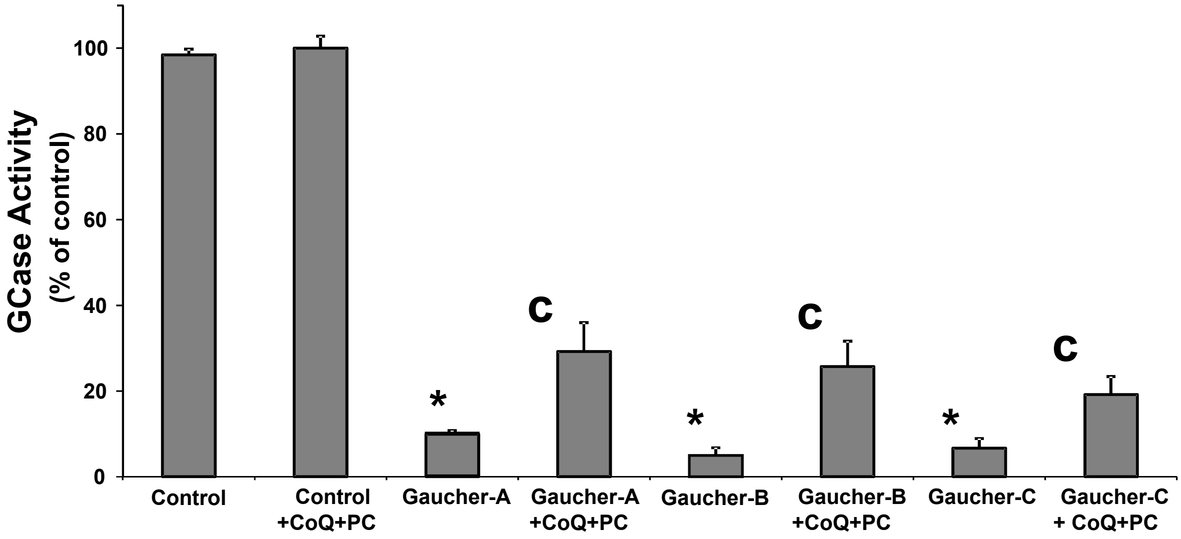

Figure S8. Treatment of Gaucher fibroblasts with CoQ and PC NAdBT-AIJ increases GCase activity. **(a)** GCase activities in Gaucher A, B and C fibroblasts. **(b)** GCase activities after CoQ+PC (25μM+25μM) supplementation in Gaucher A, B and C fibroblasts. Data represent the mean±SD of 3 separate experiments. *$p<0.01$ between control and Gaucher fibroblasts. $^{c}p<0.05$ between the presence and the absence of CoQ+PC.

a

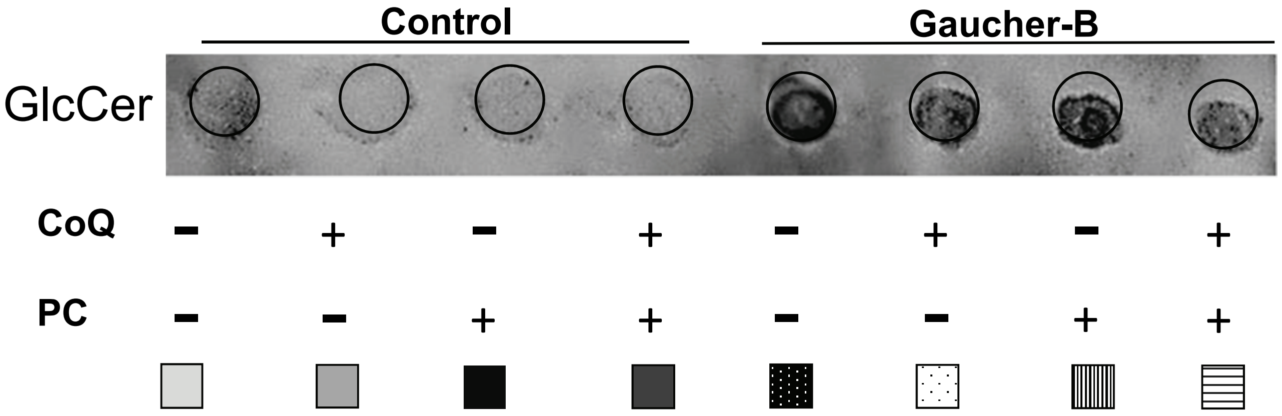

b

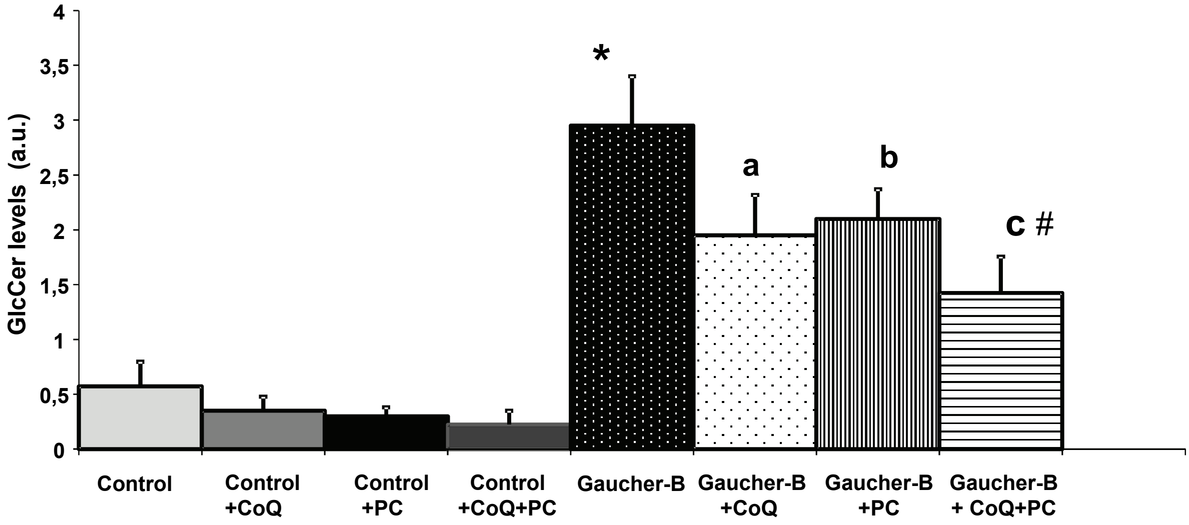

Figure S9. Treatment of Gaucher-B fibroblasts with CoQ and PC NAdBT-AIJ reduces GlcCer accumulation. **(a)** GlcCer dot-blot assay. GlcCer levels in control and Gaucher-B fibroblasts cultured in the presence or absence of CoQ (25μM), PC (25μM) or the combination of both CoQ+PC (25μM+25μM). **(b)** Densitometric analysis of GlcCer dot-blot assays. Data, expressed as arbitrary units (a.u.), represent the mean±SD of 3 separate experiments. *$p<0.01$ between control and Gaucher fibroblasts. $^{a}p<0.05$ between the presence and the absence of CoQ. $^{b}p<0.05$ between the presence and the absence of PC. $^{c}p<0.05$ between the presence and the absence of CoQ+PC. $^{\#}p<0.05$ between CoQ+PC and CoQ or PC treatment.

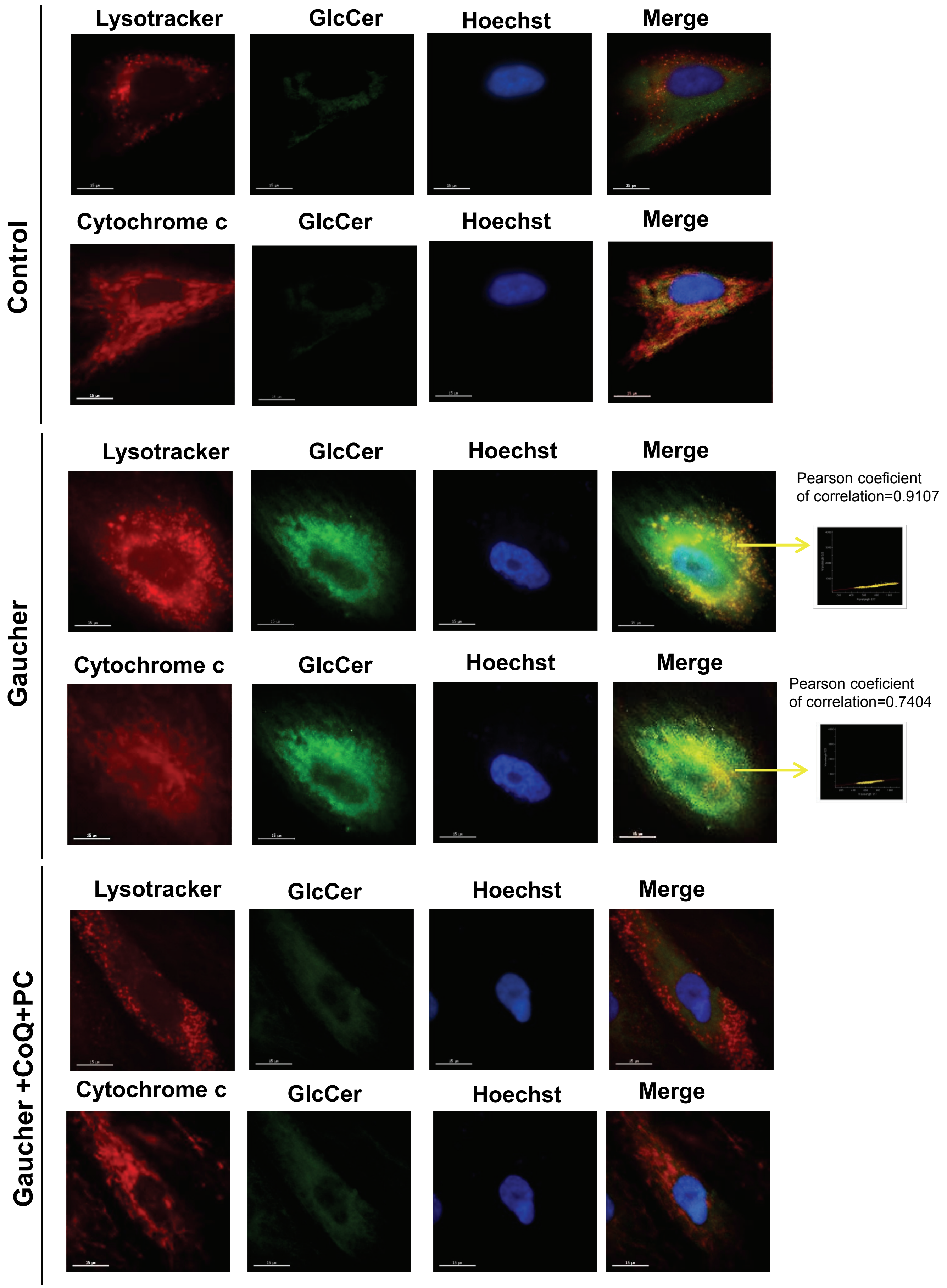

Figure S10. Effect of CoQ+PC NAdBT-AIJ on GlcCer accumulation in the lysosomal and mitochondrial compartment in Gaucher-B fibroblasts. Colocalization of GlcCer with Lysotracker or cytochrome c was assessed by calculating Pearson´s correlation coefficient by the Delta Vision software. Scale bar= 15 µm.

Figure S1. Mitochondrial function in Gaucher fibroblasts is recovered by CoQ and PC NAdBT-AIJ treatments. (a) Mitochondrial membrane potential (ΔΨm) in control and Gaucher fibroblasts was assessed by flow cytometry using the ratio TMRM/MTG signal. **(b)** Control and Gaucher fibroblasts were cultured in the absence or presence of CoQ+PC NAdBT-AIJ (25μM+25μM) for 96 h.Mitochondrial membrane potential (ΔΨm) was assessed by flow cytometry using MitoTracker Red staining. **(c)** Adenosine-5'-triphosphate (ATP) levels in control and Gaucher fibroblasts. Data represent the mean±SD of 3 separate experiments. *$p<0.01$ between control and Gaucher fibroblasts. $^{c}p<0.05$ between the presence and the absence of CoQ+PC.

Figure S2. CoQ and PC NAdBT-AIJ treatments restore tubular mitochondrial network in Gaucher fibroblasts. (a) Representative images of MitoTracker and cytochrome c staining in control and Gaucher-B fibroblasts cultured in the presence or absence of CoQ (25μM), PC NAdBT-AIJ (25μM) or the combination of both CoQ+PC (25μM+25μM). Yellow arrows indicate small depolarized mitochondria. **(b)** Quantification of depolarized mitochondria by fluorescence imaging analysis in 50 randomly selected cells from control and Gaucher fibroblasts cultures. Mitochondrial specificity of MitoTracker staining was assessed by examining colocalization of MitoTracker fluorescence with cytochrome c. Scale bar= 15 μm. Data represent the mean±SD of 3 separate experiments. *$p<0.01$ between control and Gaucher fibroblasts. $^{a}p<0.05$ between the presence and the absence of CoQ. $^{b}p<0.05$ between the presence and the absence of PC. $^{c}p<0.05$ between the presence and the absence of CoQ+PC. $^{\#}p<0.05$ between CoQ+PC and CoQ or PC treatment.

Figure S3. Effect of GlcCer accumulation on mitochondrial function. (**a**) Representative images of Mitotracker and anti-GlcCer anti-sera staining in control and Gaucher fibroblasts. Cells were incubated with 2,5mM CBE, a specific inhibitor of GCase, or 200 μM exogenous GlcCer for 24 h. Scale bar= 15 μm. (**b**) Quantification of levels of GlcCer by fluorescence imaging analysis in 50 randomly selected cells from control and Gaucher fibroblasts cultures incubated with 2,5mM CBE or 200 μM exogenous GlcCer for 24 h. Data, expressed as arbitrary units (a.u.), represent the mean±SD of 3 separate

experiments. (**C**) Quantification of ΔΨm in control and Gaucher fibroblasts incubated with 2,5mM CBE or 200 μM exogenous GlcCer for 24 h. ΔΨm was determined in individual mitochondria (n=100) from 50 randomly selected cells by fluorescence imaging analysis, as described in Materials and Methods. Data represent the mean±SD of 3 separate experiments. *p<0.01 between control and Gaucher fibroblasts. [a]p<0.01 between untreated and treated with CBE or GlcCer.

Figure S4. (a) Mitochondrial ROS levels in control and Gaucher fibroblasts cultured in the absence or presence of CoQ+PC NAdBT-AIJ (25μM+25μM) for 96 h. Results are expressed as the ratio of MitoSOX signal to 10-N-nonyl acridine orange signal. **(b)** H_2O_2 levels in control and Gaucher fibroblasts cultured in the absence or presence of CoQ+PC NAdBT-AIJ (25μM+25μM) for 96 h. H_20_2 levels were determined by CMH_2-DCFDA staining coupled with flow cytometry analysis. The mean±SD of 3 independent experiments are showed. *p<0.01 between control and Gaucher fibroblasts. [c]p<0.05 between the presence and the absence of CoQ+PC.

Figure S5. Increased expression of autophagic markers in Gaucher fibroblasts. Effect of CoQ+PC NAdBT-AIJ treatment on the amount of acidic vesicules in Gaucher fibroblasts. Control and Gaucher fibroblasts were cultured in the presence or absence of CoQ+PC (25μM+25μM) for 96 h. Acidic vacuoles were quantified LysoTracker staining and flow cytometry analysis. For control cells, the data are the mean±SD for experiments conducted on 3 different control cell lines. Data represent the mean±SD of 3 separate experiments. *p<0.01 between control and Gaucher fibroblasts. [c]p<0.05 between the presence and the absence of CoQ+PC.

Figure S6. **Densitometric analysis of Western blottings of Figure 4C.** Data represent the mean±SD of 3 separate experiments. *p<0.01 between control and Gaucher fibroblasts. [a]p<0.05 between the presence and the absence of CoQ. [b]p<0.05 between the presence and the absence of PC. [c]p<0.05 between the presence and the absence of CoQ+PC. [#]p<0.05 between CoQ+PC and CoQ or PC treatment.

Figure S7. Densitometric analysis of Western blottings of Figure 6A. Data represent the mean±SD of 3 separate experiments. *$p<0.01$ between control and Gaucher fibroblasts. $^{a}p<0.05$ between the presence and the absence of CoQ. $^{b}p<0.05$ between the presence and the absence of PC. $^{c}p<0.05$ between the presence and the absence of CoQ+PC. $^{\#}p<0.05$ between CoQ+PC and CoQ or PC treatment.

Figure S8. Treatment of Gaucher fibroblasts with CoQ and PC NAdBT-AIJ increases GCase activity. (a) GCase activities in Gaucher A, B and C fibroblasts. **(b)** GCase activities after CoQ+PC (25μM+25μM) supplementation in Gaucher A, B and C fibroblasts. GCase activities increase markedly after CoQ+PC supplementation. Data represent the mean±SD of 3 separate experiments. *$p<0.01$ between control and Gaucher fibroblasts. $^{c}p<0.05$ between the presence and the absence of CoQ+PC.

Figure S9. Treatment of Gaucher-B fibroblasts with CoQ and PC NAdBT-AIJ reduces GlcCer accumulation. (a) GlcCer dot-blot assay. GlcCer levels in control and Gaucher-B fibroblasts cultured in the presence or absence of CoQ (25μM), PC (25μM) or the combination of both CoQ+PC (25μM+25μM). **(b)** Densitometric analysis of GlcCer dot-blot assays. Data, expressed as arbitrary units (a.u.), represent the mean±SD of 3 separate experiments. *$p<0.01$ between control and Gaucher fibroblasts. $^{a}p<0.05$ between the presence and the absence of CoQ. $^{b}p<0.05$ between the presence and the absence of PC. $^{c}p<0.05$ between the presence and the absence of CoQ+PC. $^{\#}p<0.05$ between CoQ+PC and CoQ or PC treatment.

Figure S10. Effect of CoQ+PC NAdBT-AIJ on GlcCer accumulation in the lysosomal and mitochondrial compartment in Gaucher-B fibroblasts. Representative images of Lysotracker, GlcCer accumulation and cytochrome c in control and Gaucher-B fibroblasts cultured in the presence or absence of CoQ+PC NAdBT-AIJ (25μM+25μM). Colocalization of GlcCer with Lysotracker or cytochrome c was assessed by calculating Pearson´s correlation coefficient by the Delta Vision software. Scale bar= 15 μm.
